# AF9 promotes hESC neural differentiation through recruiting TET2 to neurodevelopmental gene loci for methylcytosine hydroxylation

**DOI:** 10.1038/celldisc.2015.17

**Published:** 2015-07-28

**Authors:** Yunbo Qiao, Xiongjun Wang, Ran Wang, Yuanyuan Li, Fang Yu, Xianfa Yang, Lu Song, Guoliang Xu, Y Eugene Chin, Naihe Jing

**Affiliations:** 1 State Key Laboratory of Cell Biology, Institute of Biochemistry and Cell Biology, Shanghai Institutes for Biological Sciences, Chinese Academy of Sciences, Shanghai, China; 2 Key Laboratory of Stem Cell Biology, Institute of Health Sciences, Shanghai Institutes for Biological Sciences, Chinese Academy of Sciences/Shanghai Jiao Tong University School of Medicine, Shanghai, China; 3 School of Life Science and Technology, Shanghai Tech University, Shanghai, China; 4 State Key Laboratory of Molecular Biology, Institute of Biochemistry and Cell Biology, Shanghai Institutes for Biological Sciences, Chinese Academy of Sciences, Shanghai, China

**Keywords:** AF9, TET2, human embryonic stem cell, neural differentiation, 5-hydroxymethylcytosine

## Abstract

*AF9* mutations have been implicated in human neurodevelopmental diseases and murine Af9 mediates histone methylation during cortical neuron generation. However, AF9 function and related mechanisms in human neurodevelopment remain unknown. Here we show that AF9 is necessary and sufficient for human embryonic stem cell (hESC) neural differentiation and neurodevelopmental gene activation. The 5-methylcytosine (5mC) dioxygenase TET2, which was identified in an AF9-associated protein complex, physically interacted with AF9. Both AF9 and TET2 co-localized in 5-hydroxymethylcytosine (5hmC)-positive hESC-derived neurons and were required for appropriate hESC neural differentiation. Upon binding to AAC-containing motifs, AF9 recruited TET2 to occupy the common neurodevelopmental gene loci to direct 5mC-to-5hmC conversion, which was followed by sequential activation of neural target genes and hESC neural commitment. These findings define an AF9–TET2 regulatory complex for modulating human neural development and reveal a novel mechanism by which the AF9 recognition specificity and TET2 hydroxylation activity cooperate to control neurodevelopmental gene activation.

## Introduction

The human central nervous system (CNS) is immensely complex with composition of precisely interconnected neurons. Previous studies have mainly focused on neuronal migration, subtype differentiation, and circuit formation in human neurocortex development [1–3]. The *in vitro* neural differentiation of human embryonic stem cells (hESCs) recapitulates human neural development with the presence of neural tube-like structures [4, 5]. Crucial factors, such as LIN-28 and FOXO4, have been revealed to participate in the regulation of hESC neural commitment [6, 7]. In the mouse and other animal models, multiple neurodevelopmental genes such as *ZNF521*, *SOX5*, *PAX6*, *MASH1*, *NGN2*, and *BRN2*, have been uncovered [8–12]. The transcriptional activity of these key factors largely relies on the epigenetic regulation, which has important roles in neural fate specification to guarantee the normal human neurodevelopment [13]. However, the epigenetic mechanisms underlying human neural gene activation and lineage commitment remain elusive.

AF9 (also known as MLLT3), a histone methyltransferase and MLL fusion partner, is expressed in various CNS structures and is required for mouse embryonic patterning and cerebral cortex development [14–16]. Notably, human *AF9* mutations are associated with neurodevelopmental diseases, such as mental retardation, epilepsy, and ataxia [17, 18]. Nevertheless, the function of AF9 in human neural development remains unclear. Furthermore, AF9 mediates transcriptional activation through interactions with distinct factors in different cellular processes [19–21]. AF9 also acts as an epigenetic modifier to modulate histone methylation at target gene promoters [14, 22, 23]. The report that the MLL-AF9 fusion protects *HOXA9* from DNA methylation in leukemia [24] suggests that AF9 may participate in the regulation of DNA modification. The numerous studies describing the implications of DNA methylation and hydroxylation in neural development and neurological disorders [25–27] promote us to investigate the mechanistic role of AF9-mediated DNA modification in human neural development.

TET2 is a 5-methycytosine (5mC) dioxygenase that catalyzes the conversion of 5mC to 5-hydroxymethylcytosine (5hmC) [28]. TET-mediated 5mC oxidation and DNA demethylation, which regulate gene expression and maintain cellular identity [29], are tightly correlated with neurodevelopment across species. TET1, another MLL fusion partner, promotes active DNA demethylation through hydroxylation in the mouse adult brain [30, 31]. In the mouse CNS, 5hmC is present in Purkinje neurons and the brain [32] and co-localizes with NeuN in the cerebellum to mediate epigenetic dynamics during postnatal neurodevelopment [33]. The conserved features of 5hmC in mouse brain are displayed in a 5mC demethylation activity-dependent and region-specific manner relying on TET2 activity, and 5hmC is also associated with neurodevelopmental genes in the developing human cerebellum [33–36]. Moreover, the disruption of DNA methylation-associated processes results in diverse neurological disorders [32]. In addition, 5hmC enrichment and marked TET2 upregulation during neurogenesis were observed in the fetal cortex of the human brain [37]. It has also been reported that Tet3 directly regulates key neural gene expression in the *Xenopus* eye and neural development in a dioxygenase activity-dependent manner [38]. Nonetheless, the roles of TET2 and 5hmC in human neurodevelopment are not fully understood. Importantly, how TET-mediated epigenetic regulation specifies neural gene activation and lineage commitment in human neural development remains largely unknown.

Here we show that both AF9 and TET2 are required for hESC neural differentiation. Mechanistically, AF9 physically interacts with TET2, and as a complex they bind to common neural-target gene loci to promote 5mC-to-5hmC conversion and neurodevelopmental gene activation. We further demonstrate that the TET2 occupancy at neural gene loci is guided by AF9 that recognizes AAC-containing motifs. This study provides evidence that the target specificity of TET2 is determined by the epigenetic modifier AF9 during hESC neural differentiation, thereby linking the function of AF9–TET2 complex to human neural development.

## Results

### AF9 is required for hESC neural differentiation

**Figure 1:**
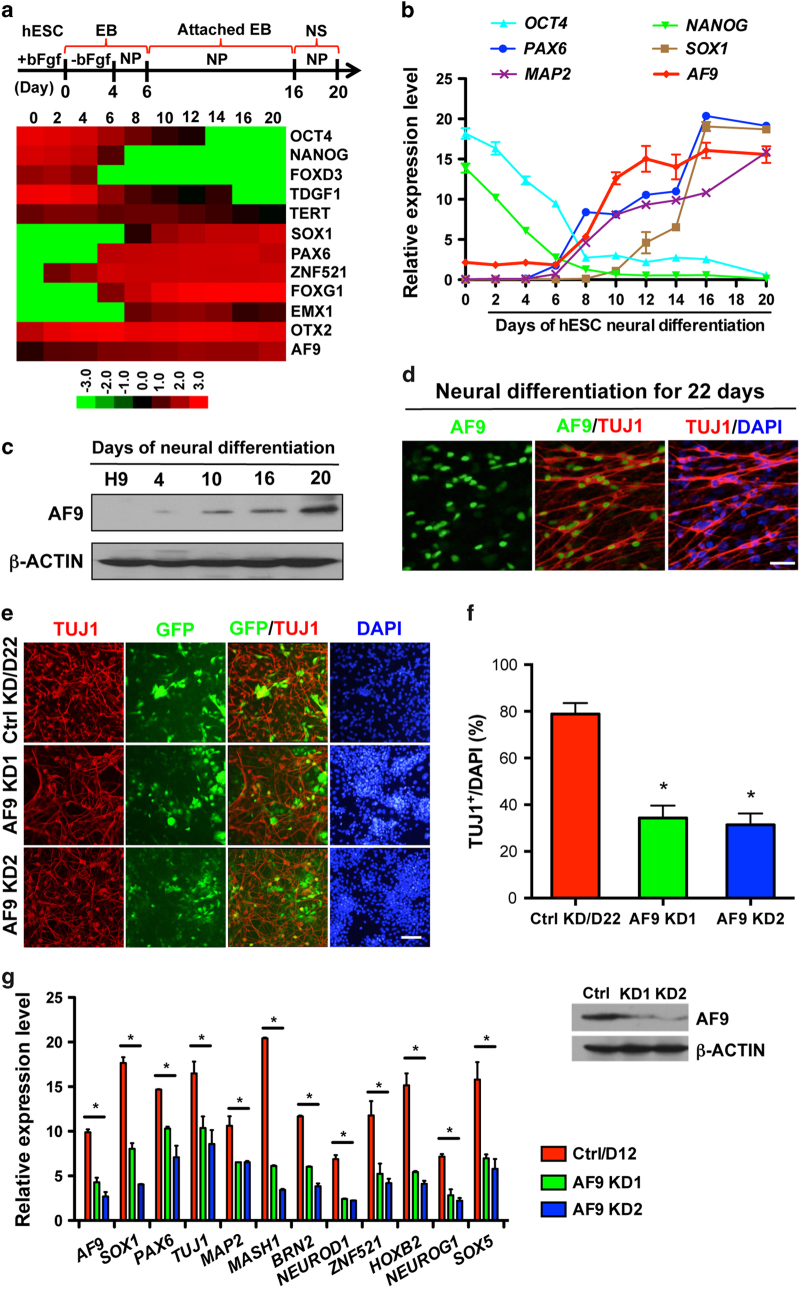
AF9 is required for human embryonic stem cells (hESCs) neural differentiation. (**a**) The upper panel shows the diagram of hESC neural differentiation procedure used in this study (see detail in experimental procedures). The lower panel shows a heat map of pluripotency genes (*OCT4*, *TDGF1*, *NANOG*, *FOXD3*, and *TERT*), early neural genes (*SOX1*, *PAX6*, and *ZFN521*), forebrain markers (*FOXG1*, *EMX1*, and *OTX2*), and *AF9* expression during hESC neural differentiation from day 0 to day 20. The log_2_-transformed normalized FPKM (fragments per kilo base per million) is assigned as expression values. (**b**) q-PCR analysis of *OCT4*, *NANOG*, *SOX1*, *PAX6*, *MAP2*, and *AF9* during hESC neural differentiation. (**c**) Western blot analysis of AF9 protein levels showing the gradual upregulation of AF9 during hESC neural differentiation. β-Actin served as a loading control. (**d**) Immunostaining of AF9 (green) and the immature neuronal marker TUJ1 (red) with their specific antibodies in day 22 differentiated hESCs. DAPI staining is shown in blue. These results show the co-localization of AF9 and TUJ1 in neuronal cells. Bar, 50 μm. (**e**) hESCs were transfected with lentivirus-mediated control shRNA (Ctrl) or AF9 shRNAs (AF9 KD1 or AF9 KD2). The three established cell lines were subjected to neural differentiation for 22 days (D22) according to the protocol described in (**a**) and these cells were stained with an anti-TUJ1 antibody. The lentiviral vector expresses green fluorescent protein (GFP) to indicate the shRNA expression status. Bar, 50 μm. (**f**) The percentage of TUJ1-positive cells was measured in control and AF9-knockdown cells. (**g**) The differentiated day 12 (D12) cells expressing control or AF9 shRNAs were collected for western blot analysis with an anti-AF9 polyclonal antibody and q-PCR analysis of the expression of AF9 and neurodevelopmental genes.

To understand the biological function of AF9 in human neural development, hESCs were induced to differentiate into neural cells as previously described (Figure 1a) [5]. During *in vitro* hESC neural differentiation, these cells underwent a temporal course with morphological features, such as the neural tube-like rosettes. To identify the neural mediators involved in this process, the global expression profile of differentiating hESCs was measured every other day using RNA-seq analysis and multiple epigenetic factors with specific expression patterns were identified (unpublished data). During the progression of neural commitment, pluripotency genes (*OCT4*, *TDGF1*, *NANOG*, *FOXD3*, and *TERT*) gradually decreased and key neural regulators (*SOX1*, *PAX6*, and *ZNF521*) and *AF9* exhibited transcriptional upregulation (Figure 1a). Consistent with previous report [5], neurons derived using this protocol expressed forebrain markers (*FOXG1*, *EMX1*, and *OTX2*) (Figure 1a). Quantitative RT-PCR (qPCR) was performed to verify the observations in RNA-seq assays. AF9 upregulation coincided with the activation of neural progenitor (NP) genes (*SOX1/5*, *ZNF521*, and *PAX6*), proneural genes (*MASH1*, *NGN2*, *NEUROG1*, and *NEUROD1/2/4*), and neuronal markers (*TUJ1* and *MAP2*) (Figure 1b; Supplementary Figure S1A). Notably, activation of these neural regulators followed a sequential neural fate determination process, and AF9 expression gradually increased from day 6 (Figure 1b), at the early stage of neural commitment [39]. Moreover, a significant increase in the protein abundance of AF9 was observed along with neural differentiation (Figure 1c), and co-immunostaining showed the specific localization of AF9 in TUJ1-positive immature neurons in differentiated cells at day 22 (Figure 1d). These findings implicate a role for AF9 in the regulation of hESC neural differentiation.

To explore its function, *AF9* was knocked down in hESCs by lentivirus-mediated *AF9*-specific shRNAs (Supplementary Figure S1B). Among infected cells, the percentages of SOX1^+^ NPs at day 12, TUJ1^+^ cells at day 22, and mature NeuN^+^ neurons at day 32 significantly decreased (Supplementary Figure S1C and S1D; Figure 1e and f). Given the low AF9 expression in hESCs (Figure 1c), depletion of AF9 did not affect its pluripotency (Supplementary Figure S1E). Moreover, AF9 knockdown does not affect the expression of other differentiating markers, which was maintained at a very low level when cells were differentiated in the neural-inducing environment (Supplementary Figure S1F), whereas the expression of multiple neurodevelopmental genes was reduced in the *AF9*-knockdown cells (Figure 1g). Clearly, disruption of neural differentiation program by *AF9* depletion suggests that AF9 is partially required for the acquisition of neural fate from pluripotent hESCs.

### AF9 overexpression promotes hESC neural commitment

**Figure 2:**
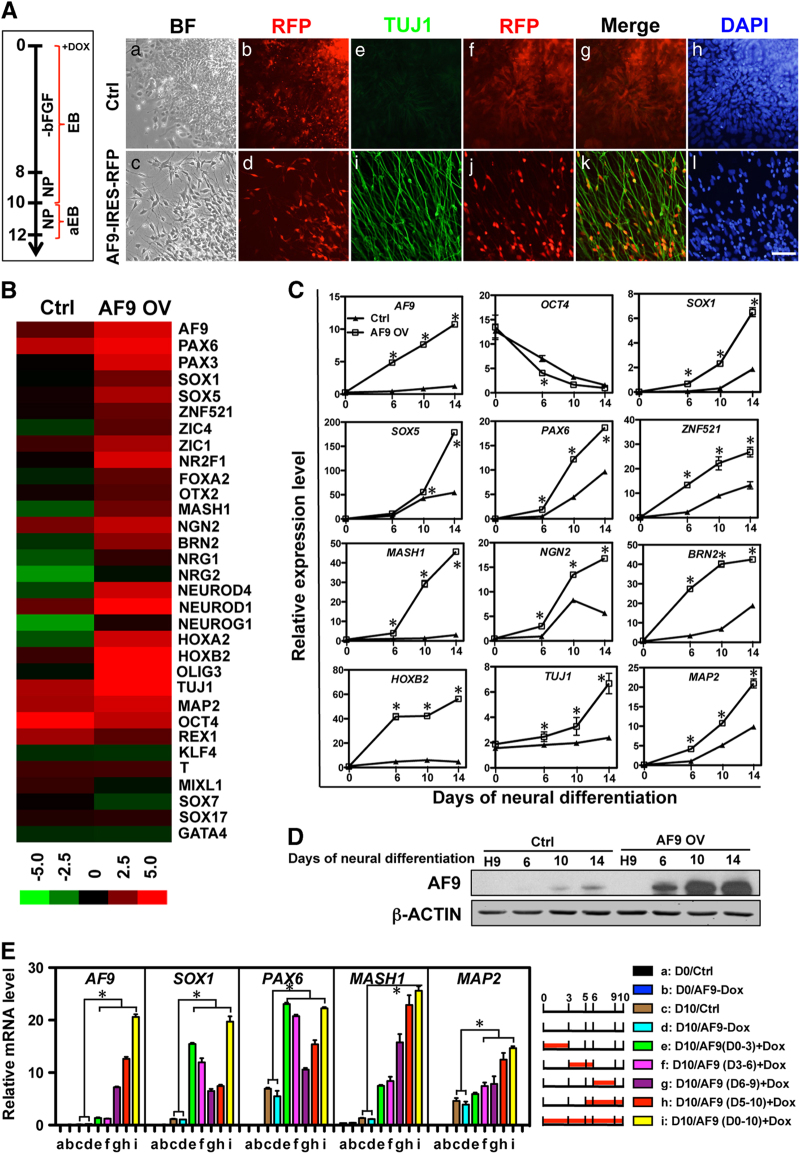
AF9 overexpression strikingly promotes human embryonic stem cell (hESC) neural differentiation. (**A**) A lentivirus-mediated control vector (Ctrl) or an inducible AF9 overexpression vector (AF9-IRES-RFP) was transfected into hESCs. The two cell lines were differentiated in bFGF-free culture medium for 8 days as EBs, and were subsequently cultured in neural progenitor (NP) medium for 2 days as EBs, followed by another 2 days as attached EBs. DOX was added to the medium throughout the entire differentiation process. Control or AF9-overexpressing cells were differentiated for 12 days, and the cell morphologies were captured (a, c) (bright feild, BF). The cells were also subjected to immunostaining with an anti-TUJ1 antibody (e, i) and counterstained with DAPI for nucleus (h, l). RFP expression represents the transfection efficiency (b, d, f ,i). The merged images of TUJ1 staining and RFP were shown (g, k). Bar, 100 μm. (**B**) Control (Ctrl) or AF9-overexpressing (AF9 OV) cells were collected for duplicated microarray analysis. Raw data were normalized by Quantile algorithm, Gene Spring Software 11.0 (Agilent technologies, Santa Clara, CA, USA). The output is a heat map of normalized signal intensity values. Multiple neural-related genes were upregulated in AF9 OV cells, while mesoendodermal markers were maintained at very low levels. (**C**) qPCR verification of the most affected genes from the microarray analysis at day 0, 6, 10, and 14, respectively. (**D**) Western blot analysis of AF9 protein levels in control (Ctrl) or AF9-overexpressing (OV) cells at the indicated time points. (**E**) AF9 overexpression was induced by doxycycline (DOX) at the indicated time intervals, and day 10 EBs were collected for the qPCR analysis of neural gene expression. Day 0 hESCs served as a control.

To address whether AF9 is sufficient in promoting hESC neural differentiation, *AF9* was cloned into a doxycycline (DOX)-inducible lentiviral expression vector containing an RFP reporter. To keep the DOX induction system stable and to make the cell counting easier, inducible AF9 lentivirus-infected hESCs were subjected to neural differentiation according to the modified protocol described in Figure 2A. In brief, lentivirus-infected hESCs were induced in the culture medium without bFGF for 8 days and in NP medium for another 2 days as embryonic bodies (EBs). Then, these EBs were attached for 2 days and subjected to immunostaining analysis with a TUJ1 antibody. AF9 overexpression was induced by DOX throughout the neural differentiation process. Comparing with the control group, overexpression of AF9 markedly enhanced the neural differentiation of hESCs with neurite outgrowth (Figure 2Aa–d). In day 12 differentiated cells, many TUJ1^+^ neurons were observed in the AF9-overexpressed cells in the marginal regions of the attached EBs, in which the neuronal marker TUJ1 was colocalized with RFP reporter reflecting AF9 expression (Figure 2Ae–i; Supplementary Figure S2A). The cells in the central region of the attached AF9-expressing EBs were mainly MASH1^+^ and a minority of these cells had differentiated into TUJ1^+^ neurons (Supplementary Figure S2A and S2B). Furthermore, partial AF9-induced neurons that migrated from EBs had differentiated into MAP2^+^ and NeuN^+^ neurons (Supplementary Figure S2C and S2D). These data suggest that AF9 overexpression accelerates the neural commitment of hESCs.

Next, we performed microarray analysis of lentivirus-infected cells at day 12 to identify AF9-activated neural genes. In total, 2 754 differentially expressed genes (1 669 upregulated and 1 085 downregulated genes) were identified. Among the top-ranking upregulated genes in AF9-overexpressing cells were a large number of neurodevelopmental genes (Figure 2B). The expression of some altered genes was confirmed by qPCR in the AF9-overexpressing cells compared with control cells. Consistent with the microarray data, the transcriptional levels of *SOX1/5*, *PAX6*, *HOXB2*, *MASH1*, *NGN2*, *BRN2*, *NEUROD1*, *TUJ1*, and *MAP2* were significantly upregulated by AF9 overexpression (Figure 2C and D). Interestingly, some hindbrain markers (*HOXA2* and *HOXB2*) were strongly induced by AF9, whereas the expression of forebrain markers (*FOXG1* and *GBX2*) was inhibited by AF9 overexpression (Figure 2B; Supplementary Figure S2E), suggesting that AF9-induced neural cells may possess hindbrain-like character and that AF9 might have a role in anterior-to-posterior patterning of the CNS. However, the expression of other germ layer markers was not affected by AF9 overexpression under the neural differentiation conditions (Supplementary Figure S2F).

In our microarray analysis, multiple neurodevelopmental genes including NP and neuronal genes were increased by AF9 overexpression. Given the sequential activation of neurodevelopmental genes during conventional hESC neural differentiation (Figure 1b; Supplementary Figure S1A), the time windows of neural gene activation by AF9 were assessed by controlling the time period of DOX treatment. The early neural markers (*PAX6* and *SOX1*) were more sensitive to induction by AF9 overexpression at the early stage of hESC neural differentiation, even though DOX-induced *AF9* transcription was reduced to the normal differentiating level at day 10 (Figure 2E). Conversely, DOX-induced AF9 at the later stage preferentially activated the proneural determinant *MASH1* and neuronal *MAP2* genes (Figure 2E). Taken together, AF9 may accelerate hESC neural commitment through activating multiple neurodevelopmental genes during hESC neural commitment.

### AF9 physically interacts with TET2

**Figure 3:**
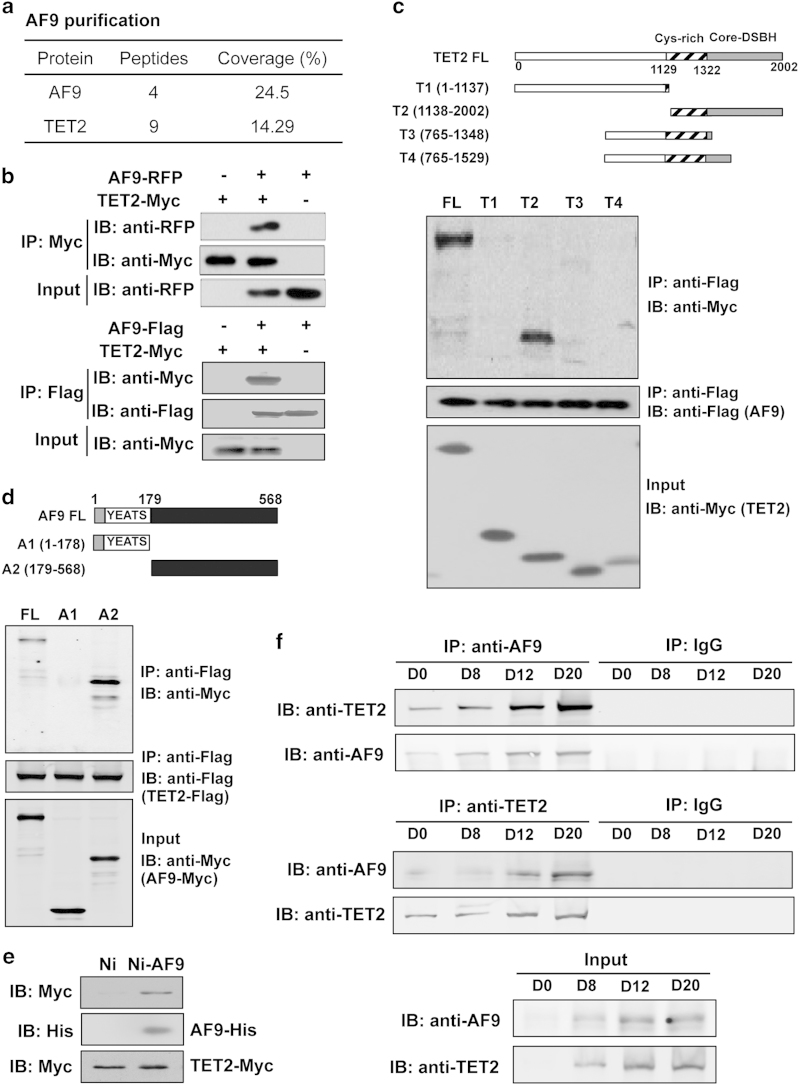
AF9 physically interacts with TET2. (**a**) Purification of AF9-associated proteins from day 12 differentiated human embryonic stem cells (hESCs). AF9-associated proteins were identified by mass spectrometry and are listed in the Supplementary Table S1. (**b**) TET2 interacts with AF9. AF9-RFP or AF9-Flag was co-expressed with TET2-myc in HEK293T cells and co-immunoprecipitations were performed with the indicated antibodies. Whole-cell lysate was used as an input. (**c**) AF9 interacts with the TET2 C-terminal domain. TET2 mutant constructs and AF9 were co-expressed in HEK293T cells. The T2 mutant containing the core-DSBH domain (catalytic domain) interacts with full-length AF9. (**d**) The C-terminal domain of AF9 interacts with TET2. Deletion mutants of AF9 mutants and full-length TET2 were co-expressed in HEK293T cells. The A2 mutant containing the C-terminal domain of AF9 interacts with TET2. (**e**) Half *in vitro* interaction between AF9 and TET2. His-tagged AF9 was purified from *E*. *coli* and incubated with cell lysate from HEK293T cells expressing TET2-Myc. (**f**) AF9 interacts with TET2 in differentiated hESCs. Cells were collected on Day 0, Day8, Day12, and Day20, respectively. IgG was used as an immunoprecipitation control; Rabbit polyclonal antibody against AF9 was used for the co-immunoprecipitation of TET2.

To identify the functional partner of AF9 in hESC neural differentiation, protein affinity purification was performed using an anti-AF9 antibody in day 12 differentiated cells, and the purified proteins were separated by sodium dodecyl sulfate–polyacrylamide gel electrophoresis (SDS–PAGE) (Supplementary Figure S3A). Mass spectrometry analysis identified 22 possible proteins including known AF9 partners, such as AF4 and HSP70 [20, 40], in the AF9-associated complex (Supplementary Table S1). AF9 modulates transcriptional activity through interacting with epigenetic regulators [19, 21]. Intriguingly, the DNA dioxygenase TET2 was present in AF9-associated complex (Figure 3a). To confirm this interaction, we performed co-immunoprecipitation assays with overexpressed AF9 and TET2 or TET1 with different fusion tags in HEK293T cells. The results showed that both TET2 and TET1 were associated with AF9 (Figure 3b; Supplementary Figure S3B).

To map the interaction regions between AF9 and TET2, we generated four TET2 deletion mutants (T1-4) and two AF9-deletion mutants (A1-2) based on their tertiary structures. We found that the carboxy-terminal catalytic domain of TET2 (TET2CD, T2) interacts with AF9 (Figure 3c) and that the carboxy-terminal domain (A2), but not the YEATS domain (A1), of AF9 was required for the interaction with TET2 (Figure 3d). Furthermore, recombinant AF9-His was purified from *E*. *coli* using nickel column chromatography, and TET2-containing lysate was obtained from TET2-myc-transfected HEK293T cells. A pull down assay revealed that TET2-myc could be co-purified from cell lysates using recombinant AF9, suggesting that AF9 tightly associates with TET2 *in vitro* (Figure 3e). To study the endogenous interaction, immunoprecipitation was performed using anti-AF9 or anti-TET2 antibodies in differentiated cells and hESCs. AF9 and TET2 exhibited a specific interaction in differentiated neural cells but not hESCs, and they showed increasing interaction strength along with hESC neural differentiation, accompanied by the increasing protein levels of AF9 and TET2 (Figure 3f). Interestingly, point mutations in the HxD motif (H1382Y and D1384A) of TET2 that are catalytically inactive [41] disrupted the AF9–TET2 interaction (Supplementary Figure S3C). Together, these data demonstrate that AF9 physically interacts with TET2 both *in vitro* and *in vivo*.

### AF9 and TET2 cooperate to promote hESC neural differentiation

**Figure 4:**
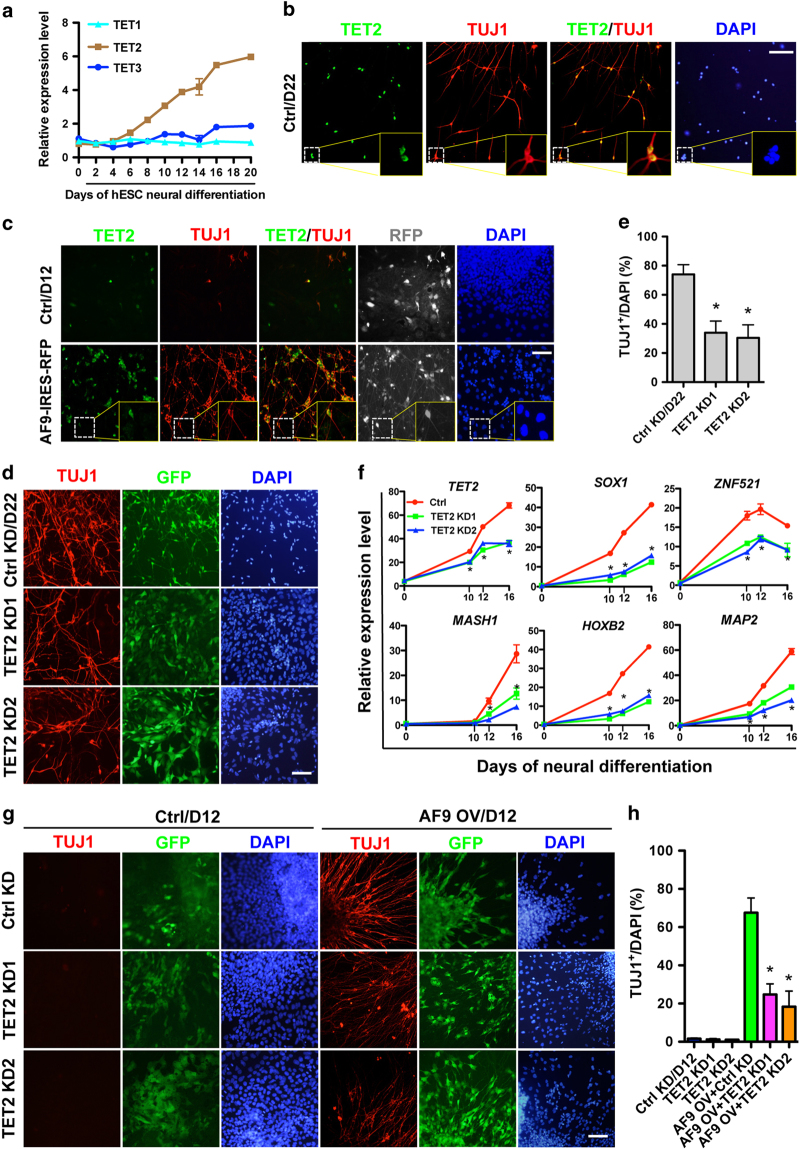
AF9 and TET2 cooperatively promote human embryonic stem cell (hESC) neural differentiation. (**a**) *TET1*, *TET2*, and *TET3* mRNA levels were determined by q-PCR during hESC neural differentiation. TET1 and TET3 were maintained at stable levels, whereas TET2 expression gradually increased during hESC neural commitment. (**b**) Neural-differentiated cells at day 22 (D22) were co-immunostained with anti-TET2 and anti-TUJ1 antibodies. The result shows that TET2 is mainly expressed in TUJ1-positive neurons. (**c**) Control (Ctrl) or AF9-overexpressing (AF9-IRES-RFP) cells were differentiated for 12 days according to the protocol in Figure 2A and co-stained with anti-TET2 and anti-TUJ1 antibodies. AF9 overexpression-induced neurons (TUJ1 positive) were also TET2 positive. (**d**) *In vitro* neural differentiation of hESCs infected with lentiviral control shRNA (Ctrl) or TET2 shRNAs (TET2 KD1 or TET2 KD2) and cultured for 22 days according to the protocol described in Figure 1a. The cells were stained with an anti-TUJ1 antibody. Green fluorescent protein (GFP) served as a marker for shRNA expression. (**e**) The percentages of TUJ1-positive cells were calculated in the control and TET2-knockdown cell populations. (**f**) The relative expression of *TET2*, *SOX1*, *MASH1*, *NEUROD2*, *HOXB2*, and *MAP2* was examined by q-PCR in control shRNA- (Ctrl) or TET2 shRNA-expressing hESCs undergoing neural differentiation. (**g**) Vector (Ctrl) or AF9-overexpressing (AF9 OV) hESCs were co-infected with lentiviral control shRNA (Ctrl KD) or TET2 shRNAs. These cells were subjected to neural differentiation for 22 days and then immunostained with an anti-TUJ1 antibody. Bars, 100 μm. (**h**) The percentages of TUJ1-positive cells in (**g**).

Given that AF9 and TET2 may form a functional complex and AF9 is required for hESC neural differentiation, we asked whether TET2 is also involved in this process. Although TET1 was also shown to interact with AF9 by co-immunoprecipitation assays in HEK293T cells (Supplementary Figure S3B), only *TET2* transcription was upregulated during hESC neural differentiation (Figure 4a), which is similar to the expression pattern of *AF9* (Figure 1b). To establish a functional connection between TET2 and neural differentiation, co-immunofluorescence was performed for TET2 and TUJ1 in day 22 cells; TET2 and TUJ1 were co-expressed in differentiated neuronal cells (Figure 4b), suggesting that TET2 might be a positive modulator of the neural differentiation process. Intriguingly, a majority of the TET2^+^ cells were TUJ1^+^ neurons induced by AF9 overexpression (Figure 4c). The activation of TET2 expression may parallel the AF9-induced progression of neural differentiation, and AF9 functions might correlate with TET2 actions.

Lentivirus-mediated knockdown assays were performed to investigate TET2 function in hESC neural commitment. Similar to AF9, *TET2* knockdown resulted in a decreased proportion of TUJ1^+^ neurons (Figurs 4d and e) and downregulation of multiple neural genes, such as *SOX1*, *ZNF521*, *MASH1*, *HOXB2*, and *MAP2* (Figure 4f). In contrast, TET2 knockdown had no significant effect on the generation of other cell lineages (Supplementary Figure S4A). Moreover, depletion of TET1 by its specific shRNA generated nearly no effect on neural differentiation (Supplementary Figure S4B), suggesting that TET1 is not the functional partner of AF9 in hESC neural commitment processes. These results demonstrate that TET2 partially contributes to human neural gene activation and hESC neural differentiation.

AF9 cooperates with distinct epigenetic factors to participate in epigenetic modifications [14, 22, 23]; we therefore asked whether AF9 and TET2 cooperatively participate in hESC neural differentiation. To test this, control or AF9-overexpressing hESCs were co-infected with TET2-specific shRNAs or control shRNA. The established hESC lines were subjected to neural differentiation for 12 days. We found that very few TUJ1^+^ cells were derived from control cells either co-expressing control shRNA or TET2 shRNAs. However, for the AF9-overexpression group, the percentage of TUJ1^+^ cells was much lower in the TET2-knockdown cells (<30%) than in the control knockdown cells (~70%) (Figure 4g and h). Consistently, the expression level of key neural regulators (*SOX5*, *NEUROG1*, *HOXB2*, *MASH1*, and *MAP2*) was decreased by TET2 knockdown in both the control and AF9-overexpressing cells (Supplementary Figure S4C), confirming the observation that AF9-induced hESC neural differentiation as impaired by TET2 depletion. Moreover, TET2 overexpression could further enhance the neural promoting activity of AF9 (Supplementary Figure S4D), and the neural promoting effect of TET2 was disrupted by catalytic negative mutations (Supplementary Figure S4E). Together, these data suggest that AF9 and TET2 cooperate to promote hESC neural commitment.

### AF9 and TET2 are enriched at the common target loci of key neural genes

**Figure 5:**
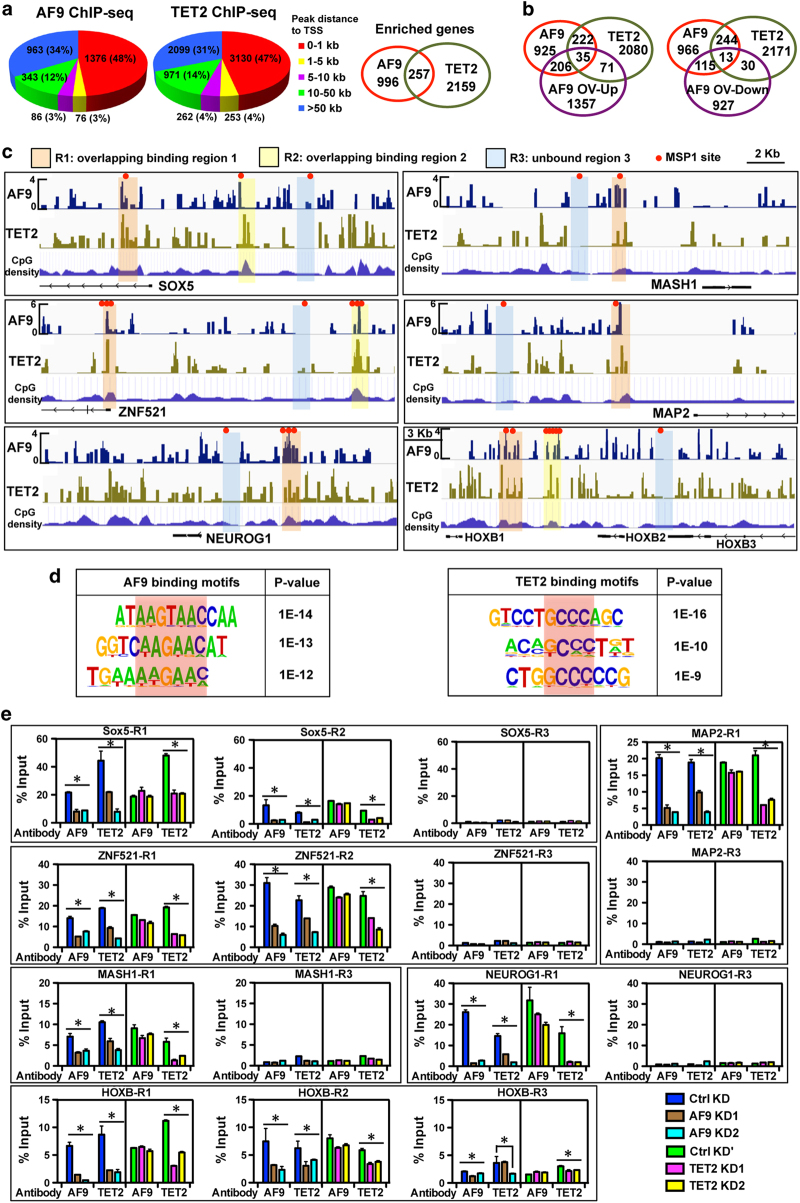
AF9 and TET2 co-occupy in neurodevelopmental gene loci. (**a**) ChIP-seq analysis of AF9 and TET2 binding sites using anti-AF9 and anti-TET2 antibodies in day 12 cells differentiated from human embryonic stem cells (hESCs). The distributions and percentages of the binding peaks are shown according to the peak distance to transcription start sites (TSSs). The number of AF9 and TET2 co-occupied genes (<5 kb) is shown in the right panel. (**b**) The AF9-enriched and TET2-enriched genes according to ChIP-seq analysis were compared with upregulated genes (left panel, AF9 OV-Up) or downregulated genes (right panel, AF9 OV-Down) upon AF9 overexpression (AF9 OV); the number of genes in each overlapping set was counted. (**c**) The binding peaks of AF9 and TET2 in the promoter regions around the transcriptional start site (TSSs) of AF9 and TET2 co-occupied genes (*SOX5*, *MASH1*, *ZNF521*, *NEUROG1*, *MAP2*, and *HOXB*) are shown. R1 and R2 represent the overlapping binding regions 1 and 2, and R3 represents the unbound region 3. The red dots represent MSP1 enzymatic sites. The lengths of displayed genomic regions are 20 kb, except for the displayed *HOXB* genomic region, which is 30 kb long. (**d**) AF9 and TET2 binding motifs and the *P-*value for each motif. (**e**) ChIP-qPCR verification of AF9 and TET2 enrichment at the R1 and R2 regions of neural target genes. The R3 region served as a negative control. R1–R3 are shown in panel (**c**).

The above findings indicated that AF9 and TET2 could cooperatively regulate key neural gene expression in hESC neural differentiation, while it remained unknown whether the AF9–TET2 complex directly regulated these neural genes. To address this question, the genome-wide distribution of AF9 and TET2 on chromatin of human neural cells was determined by chromatin immunoprecipitation sequencing analysis (ChIP-seq) using anti-AF9 and anti-TET2 antibodies, the binding specificity of which was predetermined with good yield (Supplementary Figure S5A and S5B). AF9 displayed similar binding profiles to TET2, and their binding sites are mainly located around the transcriptional start sites (TSSs) of target genes or in distal regions (>50 kb) (Figure 5a). Gene ontology analysis revealed that both AF9 and TET2 were positively involved in the regulation of neural-related cellular processes, such as neurogenesis, axonogenesis, and synapse formation (Supplementary Figure S5C). Moreover, AF9 and TET2 target genes overlapped considerably (Figure 5a), and 48 of these genes were affected by AF9 overexpression (Figure 5b).

The 35 co-occupied and AF9-activated genes included multiple key neural regulators (*SOX5*, *MASH1*, *ZNF521*, *MAP2*, *NEUROG1*, and *HOXB1-3*). AF9 and TET2 co-occupied the DNA regions around the TSSs of these genes with one (R1) or two (R1 and R2) overlapping binding regions (Figure 5c; Supplementary Figure S5D). Most occupied regions at common target genes were associated with relatively high GC density (Figure 5c). Combining the expression patterns upon AF9 overexpression, *ZNF521* and *HOXB2* were strongly enhanced at day 6 (Figure 2c), demonstrating that *ZNF521* and *HOXB2* are early AF9 target genes, while other targets might be activated at later stages. Furthermore, the essential neural regulators *PAX3/7* and *SOX-OT*, a long non-coding RNA specifically expressed in mouse brain [42], were also co-occupied by AF9 and TET2 (Supplementary Figure S5E). Interestingly, AF9 also bound to the *TET2* promoter regions (Supplementary Figure S5E), suggesting that AF9 might be an upstream factor of *TET2*. Furthermore, *de novo* motif discovery analyses were conducted to identify AF9 and TET2 binding motifs within their bound regions. Among the top ranking motifs for AF9, consensus AAC-containing elements were discovered (Figure 5d, left). Besides, C-rich sequences were found in the TET2-bound regions (Figure 5d, right) that were close to AF9-recognizing elements. Indeed, these adjacent motifs were present at the binding sites of most AF9–TET2 common target genes. Comparably, AF9–recognizing elements were much more specific than that of TET2-bound DNA sequences.

Next we validated the ChIP-seq results by using ChIP-qPCR to examine different loci in day 12 *AF9*- and *TET2*-knockdown cells (Figure 5e). In control knockdown cells, AF9 and TET2 showed strong binding activity at the R1 and/or R2 binding sites of the tested neural target genes. Remarkably, AF9 and TET2 binding activities in co-occupied regions were specifically decreased by *AF9* or *TET2* knockdown, respectively. As negative control sites, it was hard to detect the binding signals in the R3 regions (Figure 5e). More importantly, the TET2 binding activity at neural target gene loci was markedly decreased in the AF9-knockdown cells (Figure 5e), suggesting that AF9 depletion probably suppressed the recruitment of TET2 to these loci. In contrast, knockdown of TET2 did not disturb the recruitment of AF9 to the same loci (Figure 5e). Most likely, AF9 binding occurs before TET2 occupancy at the common loci, which is dependent on AF9 recruitment. Moreover, AF9-mediated TET2 targeting correlated with the expression levels of target genes (Supplementary Figure S4B). Combining the identification of AF9–TET2 interacting complex and their recognizing elements (Figure 3, Figure 5d and e), our data suggest that AF9 guides TET2 to target loci and that AF9 and TET2 might be sequentially recruited onto the regulatory regions of common downstream targets to form a complex to modulate neural gene transcription.

### 5mC conversion to 5hmC at key neural gene loci during hESC neural differentiation

We next asked how AF9–TET2 complex achieves the gene regulation. DNA methylation at gene promoters is inversely correlated with the expression of the associated genes. Tet1 and Tet2 have critical roles in 5mC oxidation to 5hmC, inducing DNA demethylation in primordial germ cells [43, 44]. Single-base resolution 5hmC mapping revealed that 5hmC was enriched within active genomic regions in mouse and human brains [35]. As TET2 could regulate hESC neural differentiation (Figure 4) and TET proteins act through the regulation of DNA methylation status [29], we investigated whether 5hmC generation was associated with the activation of TET2 target genes during hESC neural differentiation.

**Figure 6:**
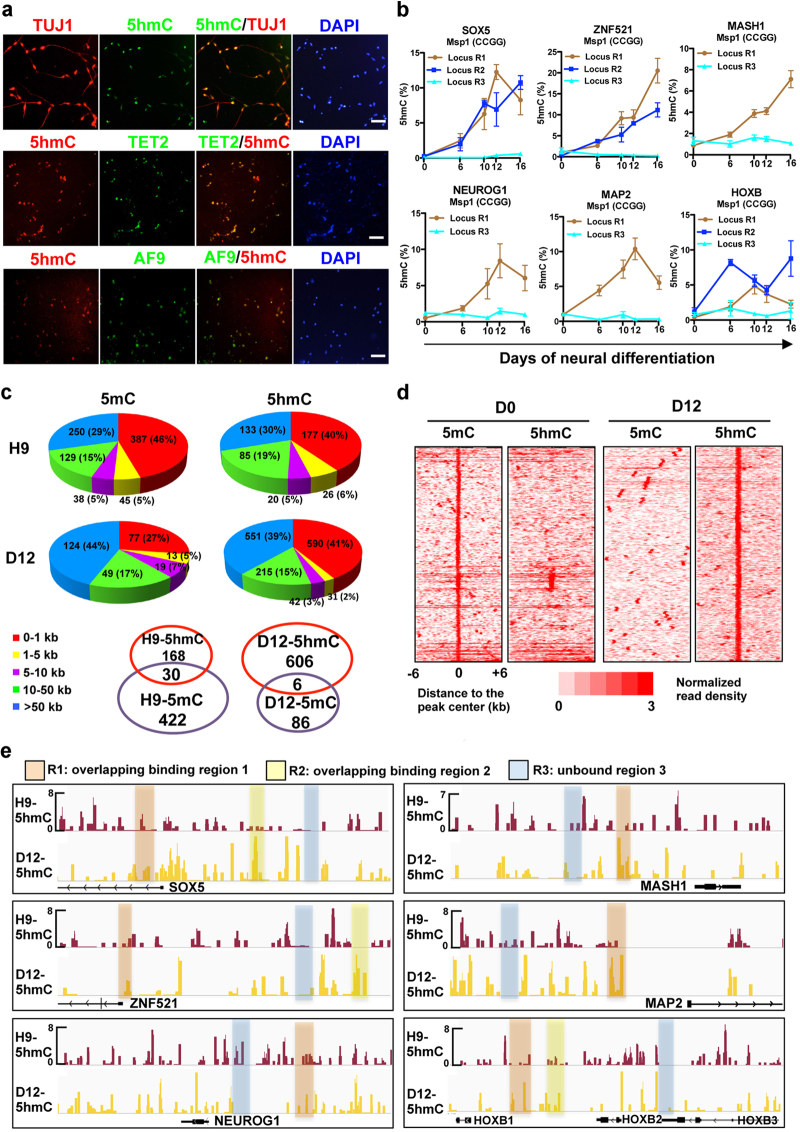
5mC conversion to 5hmC at neural gene loci during hESC neural differentiation. (**a**) The co-labeling of 5hmC with TUJ1, TET2, and AF9 in human neural cells. Cells were stained for 5hmC and co-stained for TUJ1, TET2, or AF9 in day 22 cells. Bars, 50 μm. (**b**) Glu-qPCR showing quantitative levels of 5hmC at MSP1 (CCGG) sites in the co-occupied regions of neural-related gene loci from day 0 to day 16. R1-R3 are shown as in Figure 5c. (**c**) Genome-wide distributions of 5mC and 5hmC were determined by MeDIP-seq and hMeDIP-seq. The peak distances to transcriptional start sites (TSSs) are shown. The genes that were enriched with peaks within 5 kb upstream or downstream of TSSs are defined as 5mC or 5hmC possibly regulated genes. The numbers of overlapped 5mC- and 5hmC- enriched genes (*P*-value <0.00001) in H9 and D12 cells were counted. (**d**) The global distributions of 5mC and 5hmC in day 0 and day 12 cells. The normalized read density was assigned as 5mC/5hmC levels within the regions flanking the center of 5mC/5hmC peaks. (**e**) The 5mC and 5hmC distributions within the same genomic regions of key neural genes as shown in Figure 5c.

The global 5hmC status in differentiated human neural cells was examined using co-immunostaining assays. It was found that 5hmC-positive cells were co-labeled with TUJ1, TET2, and AF9 in day 22 differentiated cells (Figure 6a), suggesting that 5hmC might be correlated with hESC neural commitment. To confirm this, glucosyltransferase-qPCR (Glu-qPCR) [43], was performed to analyze the 5hmC enrichment at neural gene loci (R1-3, Figure 5c) at different time points. We found that the 5hmC levels at the R1 and R2 loci of AF9-TET2 common target genes (*SOX5*, *ZNF521*, *MASH1*, *NEUROG1*, *MAP2*, and *HOXB*) increased during neural differentiation, while 5hmC at the control R3 loci of assessed genes remained low throughout the process (Figure 6b).

Subsequently, we performed mC/hmC-DIP-seq analysis to determine the 5mC and 5hmC levels in hESCs and day 12 differentiated cells. The 5mC and 5hmC peaks were mainly located in the proximal (within ±1 kb) and distal (> ±50 kb) regions relative to the TSS (Figure 6c), which was consistent with the observed AF9 and TET2 distributions (Figure 5a). As expected, the number of 5mC peaks in hESCs was greater than that in day 12 cells, whereas day 12 cells possessed more 5hmC peaks than hESCs (Figure 6c). The genes, in which the enrichment of 5mC/5hmC is identified within ±5 kb regions flanking TSSs, were defined as possible 5mC/5hmC-related genes. During the fate determination from hESC to neural cells, the number of 5mC-enriched genes decreased while the number of 5hmC-enriched genes increased; only a few genes enriched for both 5mC and 5hmC were observed (Figure 6c). In addition, heatmaps of 5mC/5hmC distributions (normalized read density) within the regions flanking the center of 5mC/5hmC peaks were analyzed according to the previously reported method [45]. We found that 5mC peak density markedly decreased during neural conversion from hESCs, accompanied by the remarkable increase of 5hmC peak signals in the same genomic regions (Figure 6d). It suggests that 5mC in the 5mC-enriched DNA regions in hESCs was locally converted into 5hmC during hESC neural differentiation, leading to the loss of 5mC enrichment. The gene ontology terms of 5hmC-enriched genes (active) in hESCs were highly similar to that of 5mC-enriched genes (inactive) in day 12 cells, whereas the gene ontology terms of 5hmC-enriched genes in day 12 cells were associated with neural morphogenesis-related functions (Supplementary Figure S6). Next, the 5hmC enrichment on AF9-TET2 common neural target genes was examined. Rare 5hmC peaks were observed at the loci of the analyzed genes in hESCs, and there was no 5hmC enrichment at the control R3 regions in both hESCs and day 12 cells (Figure 6e). In contrast, 5hmC peaks were newly present coincidently at the R1/R2 loci or nearby regions, which were the common binding sites of AF9 and TET2, in day 12 neural cells (Figure 6e). Together, these results suggest that 5mC is efficiently converted to 5hmC at a large cohort of genomic regions during hESC neural differentiation.

### The AF9–TET2 complex regulates 5hmC generation at neural gene loci

**Figure 7:**
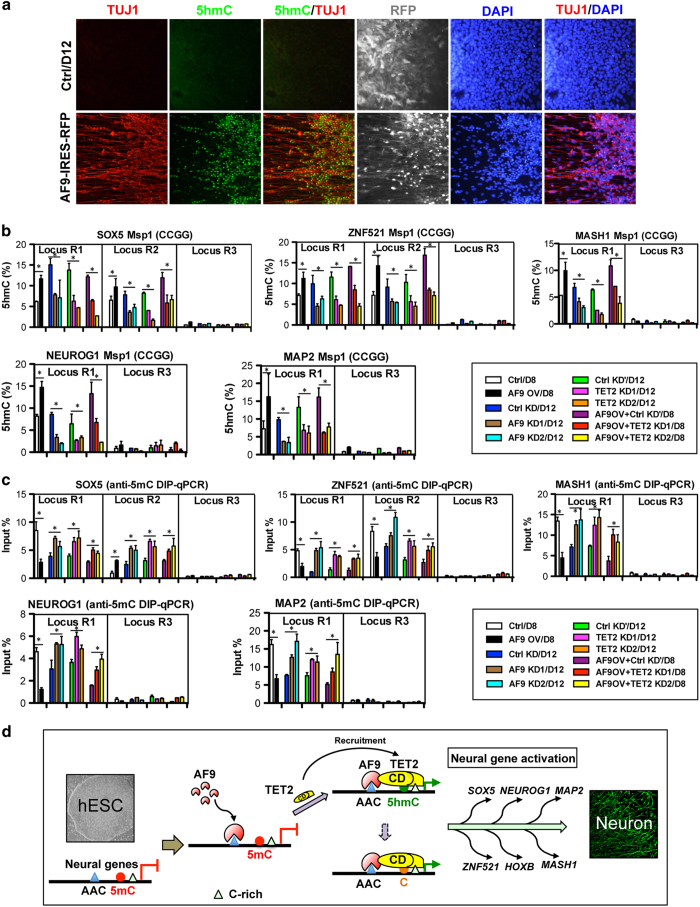
AF9 and TET2 cooperate to regulate the conversion of 5mC to 5hmC at neural gene loci. (**a**) 5hmC-positive cells were co-lobeled with TUJ1 in AF9-induced neurons. Control (Ctrl) or AF9-overexpressing (AF9-IRES-RFP) cells were differentiated for 12 days according to the protocol shown in Figure 2A and co-stained with anti-TUJ1 and anti-5hmC antibodies. Bar, 100 μm. (**b**) Glu-qPCR analysis of 5hmC levels in day 8 or day 12 differentiated cells as indicated. Ctrl/D8, day 8 cells expressing control vector; AF9 OV/D8, day 8 cells expressing AF9-IRES-RFP; Ctrl KD/D12, day 12 cells expressing control shRNA; AF9 KD1/D12 or AF9 KD2/D12, day 12 cells expressing AF9 shRNA1 or shRNA2; Ctrl KD'/D12, day 12 cells expressing control shRNA; TET2 KD1/D12 or TET2 KD2/D12, day 12 cells expressing TET2 shRNA1 or shRNA2; AF9OV+Ctrl KD’/D8, day 8 cells expressing control shRNA and AF9-IRES-RFP; AF9OV+TET2 KD1/D8 or AF9OV+TET2 KD2/D8, day 8 cells expressing TET2 shRNA1 or shRNA2 and AF9-IRES-RFP. (**c**) DNA immunoprecipitation was performed using an anti-5mC antibody. DIP-qPCR analysis was conducted to determine the 5mC levels relative to input DNA in the same group of cells in (**b**). (**d**) A model for the role of the AF9–TET2 complex in the modulation of human embryonic stem cell (hESC) neural differentiation. In hESCs, some neural gene loci are methylated and inactive. During neural differentiation, upregulated AF9 are enriched in the neurodevelopmental gene loci by recognizing the AAC-containing elements. Then, AF9 recruits TET2 to nearby C-rich DNA sequences through the TET2 catalytic domain, and the AF9–TET2 complex cooperates to direct the 5mC conversion to 5hmC or to unmethylated cytosine (**c**). The AF9–TET2 complex-mediated 5mC-to-5hmC conversions results in the activation of multiple neural genes and hESC differentiation into neurons.

A study of TET2-knockout mice proposed that DNA demethylation at 5hmC-poised loci depends on TET2 activity [35]. In day 22 differentiated cells, 5hmC was co-localized with AF9 and TET2 proteins in TUJ1^+^ neurons (Figure 6a). Having demonstrated the cooperative effect of the AF9–TET2 complex in hESC neural commitment, we next addressed whether the AF9–TET2 complex modulated 5hmC generation at their common target gene loci. Therefore, the effect of AF9 overexpression on 5hmC generation was assessed by immunostaining. We found a large number of 5hmC/TUJ1 double-positive cells after AF9 overexpression, but very few in the control experiment (Figure 7a). This result indicates that AF9 could promote 5hmC production in neural cells at the global level. At specific neural gene loci (*SOX5*, *ZNF521*, *MASH1*, *NEUROG1*, and *MAP2*), AF9 overexpression enhanced 5hmC levels while AF9 and TET2 depletion specifically decreased 5hmC distributions at the R1 and R2 regions. Moreover, AF9 overexpression-induced 5hmC increase was attenuated by TET2 knockdown (Figure 7b). In contrast, 5mC levels at these neural gene loci, detected by DIP-qPCR assays using an anti-5mC antibody, were downregulated by AF9 overexpression and upregulated by AF9 or TET2 knockdown, and the decrease of 5mC levels by AF9 overexpression was attenuated by TET2 depletion (Figure 7c). It demonstrates that the function of AF9–TET2 complex in 5mC-to-5hmC conversions at the neural gene loci depends on TET2 activity. We also found that the neural promoting effects by the overexpression of proneural gene MASH1 were partially attenuated by AF9 and TET2 double knockdown (Supplementary Figure S7), further indicating that AF9–TET2 complex accelerates hESC neural differentiation through activating their common downstream targets. Collectively, these data suggest that AF9 and TET2 regulate hESC neural differentiation through modulating 5mC-to-5hmC conversions to activate key neural genes.

## Discussion

Vertebrate neural development is coordinated by the integration of signaling pathways, key neurodevelopmental factors, and epigenetic marks. Epigenetic regulation of gene transcription is an essential and intrinsic mechanism for developmental assurance [13]. Using hESC neural differentiation as a model for human neural development, we discovered that the epigenetic factors AF9 and TET2 regulate neural development through forming a complex to directly activate the expression of key neurodevelopmental genes via an epigenetic mechanism. Similar to the function of mouse Af9 in embryonic patterning and neuron development [15, 16], human AF9 is required for hESC neural commitment through transcriptional regulation of master neural genes (Figures 1, 2 and 5). AF9 is known to modulate histone methylation by interacting with DOT1A or DOT1L [22, 46], and MLL-AF9 protects against DNA methylation of specific CpG nucleotides at the *HOXA9* gene promoter [24]. These findings suggest that AF9 may act as a co-factor to coordinate the enzymatic activities of other proteins in epigenetic modifications. Our mechanistic study identifies AF9 as an active regulator of DNA hydroxylation, through interacting with TET2 and guiding TET2 to specific neural gene loci in neurodevelopment, resulting in the 5mC-to-5hmC conversions and neural gene activation. Although Af9 has been implicated in cortical development through mediating H3K79 dimethylation, the functional effect of Af9 in this context exclusively relies on its associated partner-Dot1L [14], which was expressed in both hESCs and NPCs at stable levels (data not shown) and was not detectable in AF9-associated complex (Supplementary Table S1). It suggests that AF9-mediated histone methylation might not be involved here and that TET2 is the specific functional partner of AF9 in hESC neural differentiation through modulating DNA hydroxylation.

TET family proteins and their catalyzed product 5hmC have been extensively associated with neurodevelopment. *Xenopus* Tet3 directly activates master neural genes (*rx*, *pax6*, and *ngn2*) in eye and neural development during embryogenesis [38]. Murine Tet1 regulates hippocampal neurogenesis and lineage specification, and promotes active DNA demethylation in the adult brain and mouse ESCs (mESC) [30, 31, 47, 48]. Recently, it has been reported that mouse Tet3 is important for mESC neural differentiation [49]. Although Tet1- and Tet2-knockout mice develop normally [50, 51], Tet3 deletion leads to neonatal lethality [52]. Here we reveal TET2 as a necessary factor for early key neural gene activation in hESC neural differentiation (Figures 4 and 7). These findings highlight a convergent role of TET proteins in neurodevelopment across species, and suggest that specific TET family members may be responsible for different developmental processes in different species [53].

TET family possesses only three members but catalyze DNA hydroxylation in the whole genome in a tissue-specific manner, suggesting specific mechanisms of their target specificities. It has been reported that the N-terminal CXXC domain of *Xenopus* Tet3 is required for DNA binding specificity with a slight preference for CpG content [38]. We also consistently reveal that human TET2, which lacks the CXXC domain, occupies at a C-rich element (Figure 5), while the binding sequences of both TET3 and TET2 are not specific enough for TET2/3 targeting to specific neural gene loci. In agreement with previous hypothesis, TET DNA binding and enzymatic activity may be coordinated with its associated cofactors to modulate gene transcription [38]. This hypothesis is supported by the reports that neuronal Tet3 is recruited by REST for induction of target gene expression [54] and that TET enzymatic activation depends on interaction with CRL4 complex in primordial follicles [55]. Here we show that AF9 physically interacts with TET2 *in vivo* and *in* *vitro* (Figure 3) and that they collaborate to regulate 5hmC generation and activate neurodevelopmental genes (*SOX5*, *ZNF521*, *MASH1*, *NEUROG1*, and *MAP2*) (Figures 6 and 7). Both AF9 and TET2 are MLL fusion partners that regulate *HOX* gene transcription in hematological malignancies [56–58]. ChIP-seq and ChIP analysis revealed that AF9 and TET2 co-occupied the common neural-specific gene loci and that TET2 enrichment at these sites depends on AF9 recruitment (Figure 5). Functionally, the neural-promoting effect of AF9 is dependent on TET2 ability to promote 5mC-to-5hmC conversions at neurodevelopmental gene loci (Figures 4 and 7). This result is consistent with the role of TET2 in maintaining *HOXA* activity through 5mC hydroxylation at lineage-specific loci [59].

DNA methylation dynamics are associated with brain development, maturation and synaptogenesis [35, 60]. Consistently, a global epigenomic transition from 5mC enrichment in hESCs to 5hmC enrichment in neural cells was observed, correlating with neurogenesis and synapse formation (Figure 6; Supplementary Figure S6). 5hmC marks brain cell genomes in the fetal and adult brain [30, 31, 35, 37] and TET3-mediated 5hmC generation is an important epigenetic mechanism for master neural gene activation in *Xenopus* [38]. Importantly, CG demethylation at 5hmC-poised loci in the genomes of mouse brain cells depends on Tet2 activity, and both 5hmC and TET2 have been observed in the human brain cortex [26, 35, 37]. These observations support our findings that TET2 has positive roles in hESC neural commitment through directing 5mC hydroxylation at neural-specific gene loci (Figures 4 and 7). The C-terminal catalytic domain of human TET2 is responsible for DNA hydroxylation, DNA binding [61], and interaction with AF9 (Figure 3). Our study reveals a novel aspect of TET2 activity in human neural development, which relies on complex formation with AF9 that is enriched in sequence-specific motifs in neural-specific gene loci and provides recognition and recruitment signals for TET2. The specific expression of AF9 and TET2 during hESC neural differentiation is crucial for the establishment of an appropriate DNA hydroxylation profile and allow progressive neural commitment. Interestingly, the catalytic domain of TET2, which is important for overall structure formation of TET2 and TET2-DNA interactions [61], is important for its association with AF9 (Supplementary Figure S3C). It is possible that HXD motif mutation, which abolished enzymatic activity [61], may lead to the protein conformation change of C-terminal domain of TET2.

In addition, the striking effect of AF9 in promoting neuronal differentiation (Figure 2) was similar to the single-step induction of neurons observed upon forced expression of master neural regulators, such as MASH1 and NGN2 [62, 63]. The main difference is that the AF9–TET2 complex sequentially activates the expression of endogenous NP genes, proneural genes and neuronal genes through epigenetic modulation of the 5mC/5hmC status. Interference of DNA methylation disrupted the neural gene activation program (Supplementary Figure S1A and S6), indicating the importance of the AF9–TET2 complex for maintaining the well-balanced DNA modification profiles. In agreement with this notion, *AF9* mutations and disturbed DNA methylation or hydroxymethylation have been implicated in human neurodevelopmental disorders [18, 34, 64]. Our study provides an explanation why these genetic and epigenetic abnormalities may result in abnormal neural gene transcription and neurodevelopment.

In summary, our findings reveal a molecular model for the role of AF9–TET2 complex in neurodevelopment. This model involves the targeted recruitment of TET2 to 5mC-containing genomic loci by AF9, followed by TET2-mediated hydroxylation that converts 5mC to 5hmC at neural-specific gene loci (Figure 7d). AF9 occupies the neurodevelopmental gene loci via recognizing motifs containing AAC nucleotides and, with its C-terminal region (Figure 3), recruits TET2 to the adjacent C-rich regions at the loci. The recruited TET2 catalyzes the conversion of CD-bound 5mC to 5hmC during hESC neural differentiation and subsequently activates target gene expression to ensure normal and precisely controlled human neural development.

## Materials and Methods

### Cell culture and treatment

H9 hESC maintenance and neural differentiation were performed as previously described [5]. In brief, normally cultured hESCs in standard medium with bFgf were transferred to bFgf-free medium as aggregates (EBs) for 4 days. The EBs were then cultured in NP medium for another 2 days. Subsequently, the EBs were attached to the dish (called attached EB) and cultured in NP medium for 10 days. Then, the attached EBs were dissociated into patches and cultured as suspended neural spheres for 4 days. Finally, the neural spheres (NS) were trypsinized to single cells and replated onto culture dishes. HEK293T cells were cultured in DMEM medium supplemented with 10% FBS.

### Immunoprecipitation and co-immunoprecipitation

Immunoprecipitation and co-immunoprecipitation assays were performed according to our previously described protocol [65]. The following antibodies were used: anti-AF9 (NB100-1566, Novus Biologicals, Littleton, CO, USA), anti-Myc (Santa Cruz Biotechnology, Santa Cruz, CA, USA), and anti-Flag (Sigma, St Louis, MO, USA).

### Mass spectrometry

The immunoprecipitation assay was performed as described above using day 12 cells differentiated from hESCs and anti-AF9 antibodies (NB100-1566, Novus Biologicals). The AF9-associated protein bands were subjected to in-gel digestion and mass spectrometric characterization [65] (Thermo Fisher Scientific, Waltham, MA, USA). The identified proteins are listed in Supplementary Table S1.

### Gene overexpression and knockdown in hESCs

Lentivirus-mediated gene overexpression and knockdown in human H9 cells were performed as previously described [66], and the lentiviral infection method used was described in a published protocol [67]. Lentiviral infected clones were enriched by manual selection or sorted by FACS. The control and shRNA sequences are listed in Supplementary Table S2.

### RNA extraction and qPCR analysis

Total RNA was extracted from cultured cells using TRIzol reagent (Pufei Bio, Shanghai, China). Reverse transcription and qPCR were performed as previously described [68]. All qPCR results were repeated for at least three times and one representative result was shown. The primers are listed in Supplementary Table S3.

### Half *in vitro* interaction assay

His-AF9 was purified from BL21 (DE3) cells, cross-linked with Ni-agarose, and then incubated for 4 h at 4 °C with lysate from HEK293T cells expressing TET2-myc. After incubation and wash, the Ni-agarose was boiled at 100 °C for 10 min, and the eluted samples were loaded for SDS–PAGE analysis.

### ChIP-seq

ChIP was performed as previously described [68] using anti-AF9 (NB100-1566, Novus Biologicals) and anti-TET2 (R1086-1b, Abiocode, Agoura Hills, CA, USA) antibodies with day 12 cells (GEO: GSE68332). The high-throughput sequencing was performed by the Computational Biology Omics Core (CAS-MPG Partner Institute for Computational Biology, China).

### MeDIP/hMeDIP-seq and DIP-qPCR

Genomic DNA was purified from hESCs and day 12 differentiated cells and sonicated. MeDIP and hMeDIP were performed as previously described [47] using anti-5mC (39649, Active Motif, Carlsbad, CA, USA) or anti-5hmC antibodies (MABE317, Millipore, Darmstadt, Germany) (GEO: GSE68396). The immunoprecipitated DNA was sequenced by the Computational Biology Omics Core (CAS-MPG Partner Institute for Computational Biology, China). To detect the 5mC levels, the immunoprecipitated DNA using an anti-5mC antibody and input DNA were subjected to qPCR analysis. Then input % was calculated and assigned as the relative 5mC level.

### Glu-qPCR

The quantitative level of 5hmC was determined by Glu-qPCR [43] using the Quest 5-hmC Detection Kit (Zymo Research, Orange, CA, USA) according to the manufacturer’s instructions. The primers used for ChIP verification and Glu-qPCR are listed in Supplementary Table S4. The primers used for ChIP verification, DIP-qPCR and Glu-qPCR are listed in Supplementary Table S4.
