# Supplementary material for: AF9 promotes hESC neural differentiation through recruiting TET2 to neurodevelopmental gene loci for methylcytosine hydroxylation
Source: Cell Discov. 2015 Jul 28;1:15017–. doi: 10.1038/celldisc.2015.17 (PMC4860857; doi:10.1038/celldisc.2015.17)
Supplement: Supplementary Information [file celldisc201517-s1.doc]

**Supplementary Figure Legends**

**Figure S1. AF9 knockdown in hESCs.**

**(A)** The relative expression levels of multiple neurodevelopmental genes were examined by qPCR during hESC neural differentiation.

**(B)** The infection efficiency of control (Ctrl KD) and AF9 knockdown (AF9 KD1 and KD2) lentiviruses in hESCs. Scale bar, 200 μm.

**(C)** Immunostaining of day 12 differentiated control or AF9 knockdown cells was performed using an anti-SOX1 antibody. Scale bar, 100 μm. The percentages of SOX1-positive cells were calculated.

**(D)** D32 differentiated control or AF9 knockdown cells were subjected to immunostaining using an anti-NeuN antibody. Scale bar, 100 μm. The percentages of NeuN-positive cells were calculated.

**(E)** The pluripotency of AF9-knockdown hESCs (H9) was not altered based on the examination of *OCT4*, *NANOG*, and *GDF3* expression.

**(F)** The expression of pluripotency or differentiation markers was determined in day 12 cells expressing control (Ctrl KD) and AF9 shRNAs (AF9 KD1 and KD2).

**Figure S2. AF9 overexpression in hESC neural differentiation.**

**(A)** D12 cells expressing the control (Ctrl) or AF9 overexpression vector (AF9-IRES-RFP) were immunostained with an anti-TUJ1 antibody. RFP expression represents the infection efficiency. Scale bar, 500 μm.

**(B)** Control (Ctrl) or AF9-overexpressing (AF9 OV) cells were subjected to double immunostaining with a polyclonal anti-MASH1 antibody and a monoclonal anti-TUJ1 antibody. Scale bar, 200 μm.

**(C)** Co-immunostaining of TUJ1 and MAP2 in day 14 differentiated hESCs infected with control (Ctrl) or AF9 overexpression vector (AF9-IRES-RFP). Scale bar, 100 μm.

**(D)** Immunostaining of NeuN in day 14 differentiated hESCs infected with control (Ctrl) or AF9 overexpression vector (AF9-IRES-RFP). Scale bar, 100 μm.

**(E)** Normalized expression level of forebrain and hindbrain markers from microarray analysis in Figure 2B.

**(F)** qPCR analysis of the relative expression levels of pluripotency or differentiation markers in day 12 cells expressing the control (Ctrl) or AF9 overexpression vector (AF9 OV).

**Figure S3. AF9 interacts with TET proteins.**

**(A)** AF9-associated proteins were purified from day 12 differentiated hESCs using an anti-AF9 antibody and separated in a 5-15% gradient SDS-PAGE gel. Normal IgG served as a control.

**(B)** AF9 interacts with TET1. AF9-RFP was co-expressed with TET1-Myc in HEK293T cells and co-immunoprecipitation was performed with an anti-Myc antibody.

**(C)** AF9 interaction with TET2 depends on TET2 enzymatic activity. TET2-myc containing indicated mutations and AF9-Flag were co-expressed in HEK293T cells and co-immunoprecipitation was conducted with an anti-Flag antibody.

**Figure S4. Promotion of neural gene expression by AF9 depends on TET2 cooperation.**

**(A)** The expression of pluripotency or differentiation markers was examined in differentiated hESCs expressing the control (Ctrl KD) or TET2 shRNAs (TET2 KD1 and TET2 KD2).

**(B)** TET1 shRNA was constructed into modified inducible plvut-tTR-KRAB vector (H1 promoter-driven). hESCs were infected by lentiviruses containing control (Ctrl KD) or TET1 shRNAs and subjected to neural differentiation with DOX treatment. Day 12 differentiated cells were collected for qPCR analysis of pluripotency and neural gene expression.

**(C)** Empty vector- (Ctrl) or AF9-overexpressing (AF9OV) hESCs were co-infected with control shRNA (Ctrl KD) or TET2 shRNA (TET2 KD1 and TET2 KD2) lentiviruses. These cells were subjected to neural differentiation for 22 days and then cells were collected for qPCR analysis of *AF9*, *TET2*, *SOX5*, *NEUROG1*, *HOXB2*, *MASH1*, and *MAP2* expression.

**(D)** hESCs were infected with empty vector (Ctrl), AF9-overexpression (AF9 OV), TET2 overexpression (TET2 OV), or combination of AF9 OV and TET2 OV (AF9OV+TET2 OV) lentiviruses. These cells were subjected to neural differentiation for 8 days and then these cells were collected for qPCR analysis of *AF9*, *TET2*, *SOX5*, *HOXB2*, *MASH1*, and *MAP2* expression.

**(E)** hESCs were infected with empty vector (Ctrl), TET2 overexpression (TET2 OV), or mutated TET2 (H1382Y and D1384A) lentiviruses (TET2 point mutation, TET2 PM). The differentiated cells were subjected to qPCR analysis of neural gene expression.

**Figure S5. AF9 and TET2 co-occupy in neural target gene loci.**

**(A-B)** The immunoprecipitation efficiency of the AF9 antibody (A) and TET2 antibody (B) was measured in day 12 differentiated hESCs with the indicated amount of antibodies. The samples in (B) were separated in a 5-15% gradient gel. Normal IgG was used to normalize the amount of antibody in each reaction.

**(C)** The input signal for the ChIP-seq analysis described in Figure 5C.

**(D)** The binding activities of AF9 and TET2 at the promoter regions around the TSSs of co-occupied genes (*PAX7, PAX3,* and *SOX2-OT*) are shown. The brown labeled regions represent AF9 and TET2 co-occupied regions. The lower panel shows the AF9 enrichment at the TET2 promoter. The length of the displayed genomic regions is 20 kb.

**(E)** The GO-analysis of AF9- and TET2-enriched genes in hESC-derived neural cells.

**Figure S6. The conversion of 5mC to 5hmC during hESC neural commitment.**

GO analysis of 5mC- and 5hmC-enriched genes in hESCs and day 12 differentiated cells. The 5hmC-enriched genes represent activated genes, and the 5mC-enriched genes represent inactivated genes.

**Figure S7. The neural promotion effect of MASH1 is partially affected by AF9 and TET2 knockdown.**

hESCs were infected with control vector (Ctrl), combination of AF9 shRNA1 and TET2 shRNA1 (AF9 KD+TET2KD), MASH1 overexpression (MASH1 OV), or combination of AF9 shRNA1, TET2 shRNA1, and MASH1 overexpression (AF9 KD+TET2 KD+MASH1 OV) lentiviruses. These cells were subjected to neural differentiation for 12 days and then these cells were collected for qPCR analysis of *MASH1*, *AF9*, *TET2*, PAX6, *SOX5*, *ZNF521*, *HOXB2*, and *MAP2* expression.

**Supplemental inventory:**

Figure S1, related to Figure 1 in paper

Figure S2, related to Figure 2 in paper

Figure S3, related to Figure 3 in paper

Figure S4, related to Figure 4 in paper

Figure S5, related to Figure 5 in paper

Figure S6, related to Figure 6 in paper

Figure S7, related to Figure 7 in paper

Table S1, related to Figure 3 in paper

Table S2, related to Figure 1-5 in paper

Table S3, related to Figure 1-5 in paper

Table S4, related to Figure 5-7 in paper
