# Supplementary figures and images for: AF9 promotes hESC neural differentiation through recruiting TET2 to neurodevelopmental gene loci for methylcytosine hydroxylation
Source: Cell Discov. 2015 Jul 28;1:15017–. doi: 10.1038/celldisc.2015.17 (PMC4860857; doi:10.1038/celldisc.2015.17)

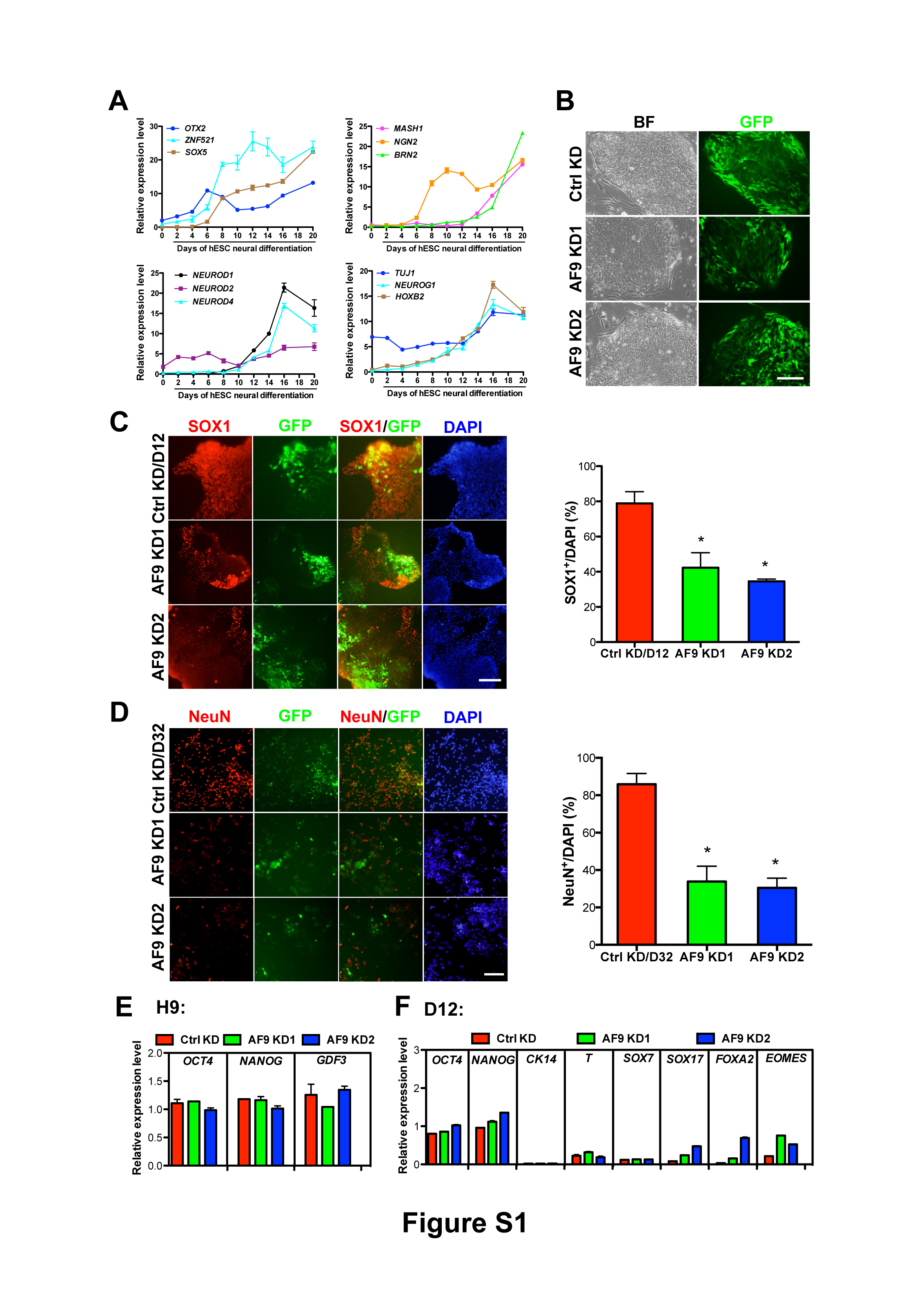

Supplement: Supplementary Figure S1 [file celldisc201517-s2.jpg]

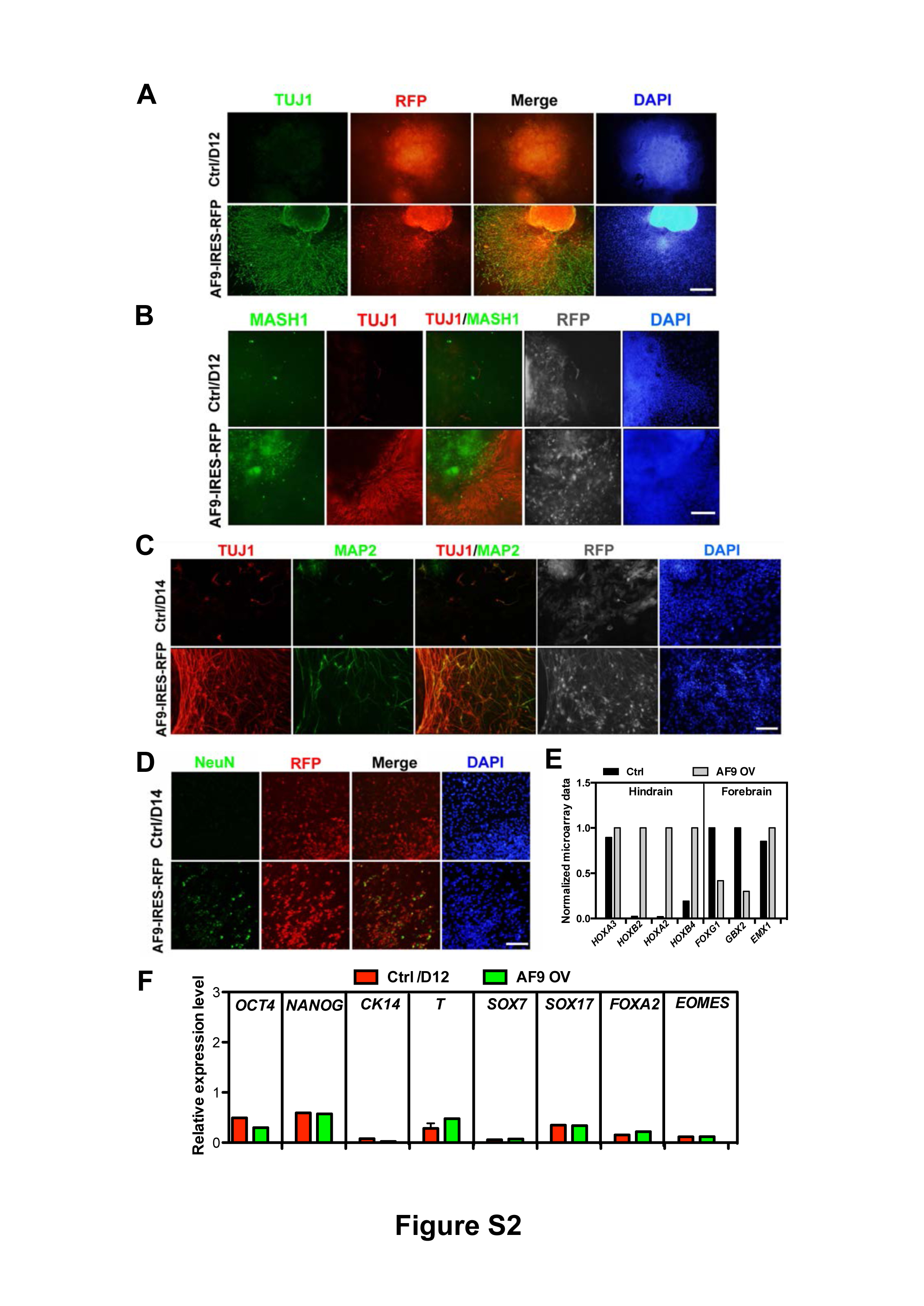

Supplement: Supplementary Figure S2 [file celldisc201517-s3.jpg]

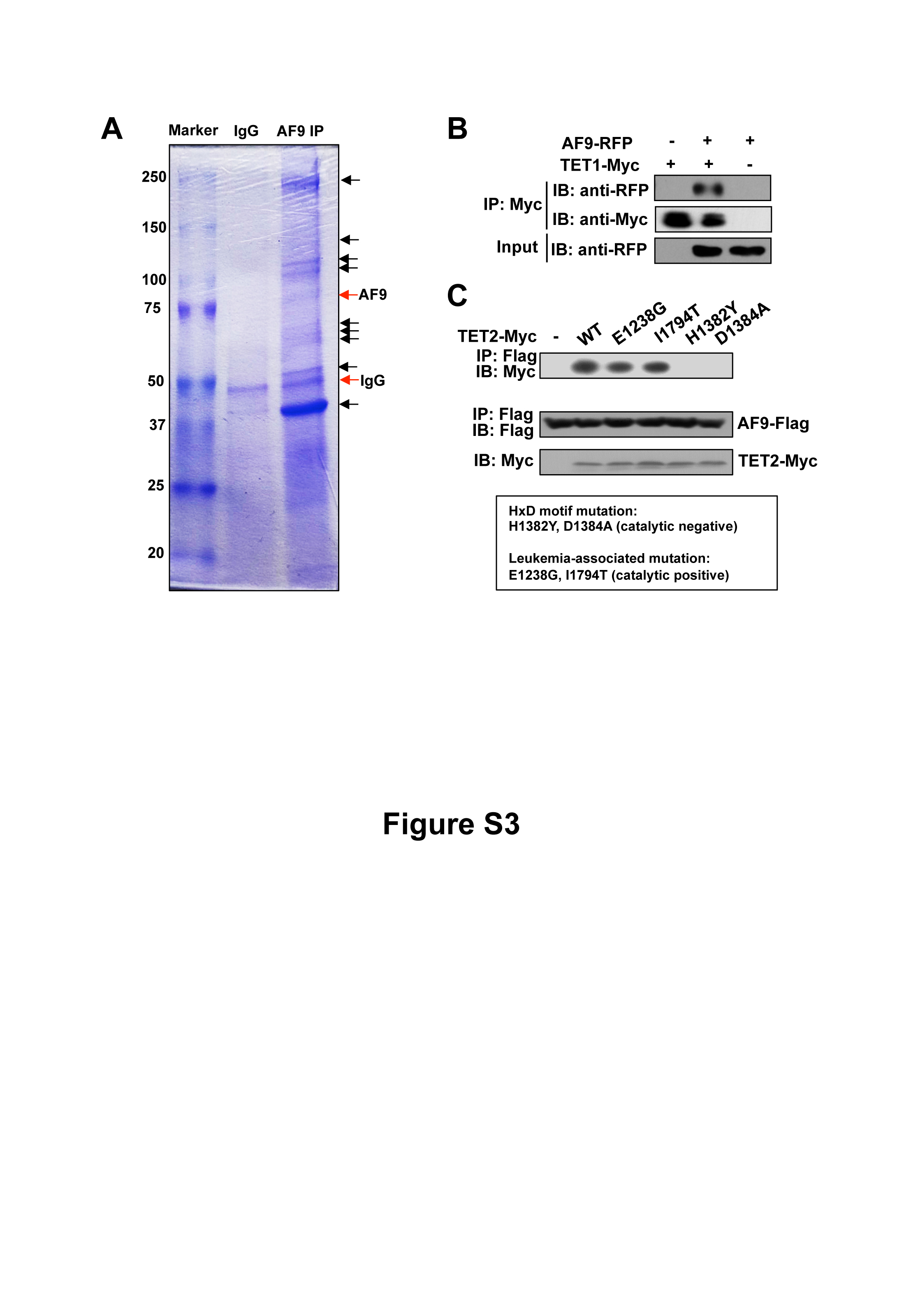

Supplement: Supplementary Figure S3 [file celldisc201517-s4.jpg]

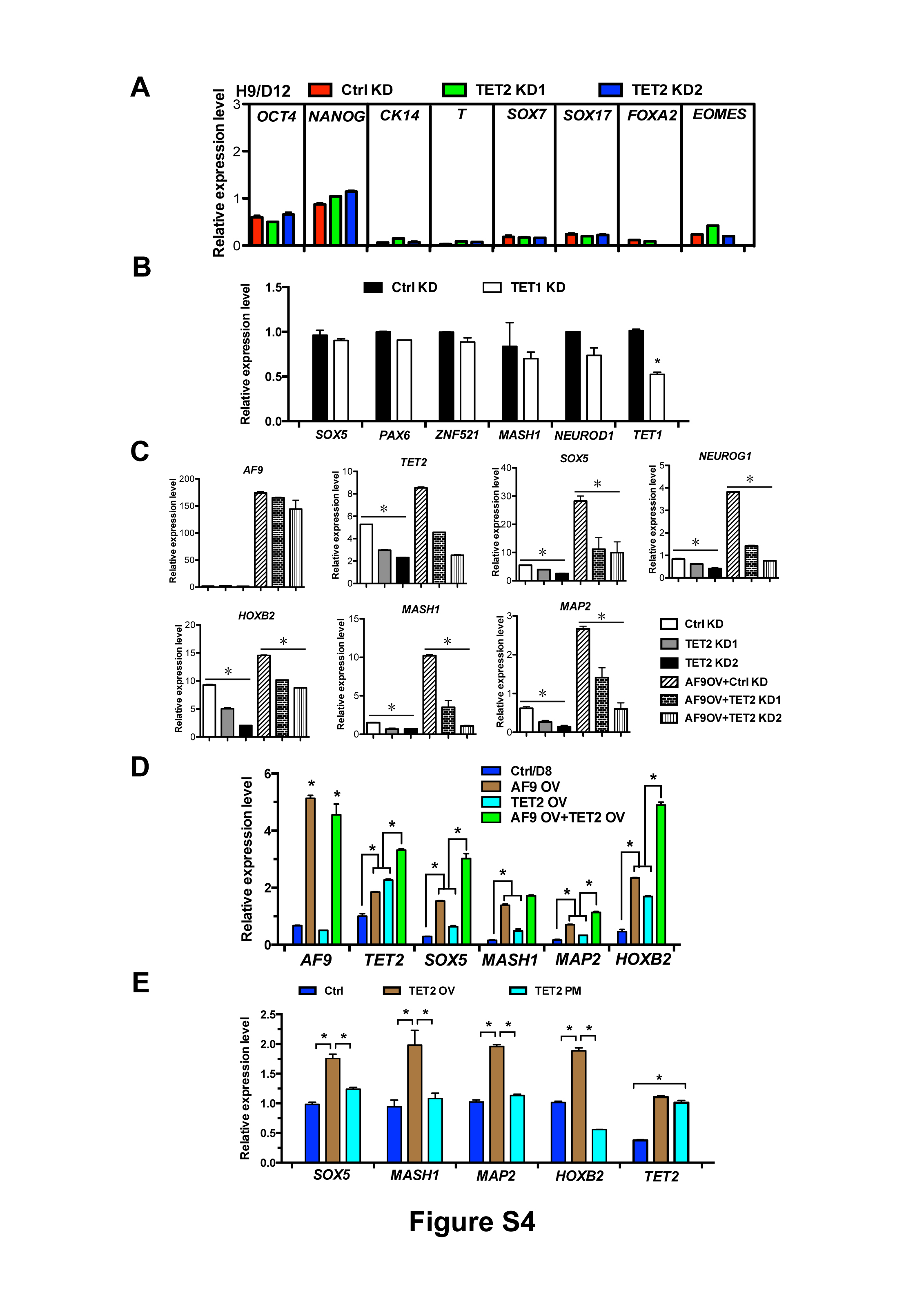

Supplement: Supplementary Figure S4 [file celldisc201517-s5.jpg]

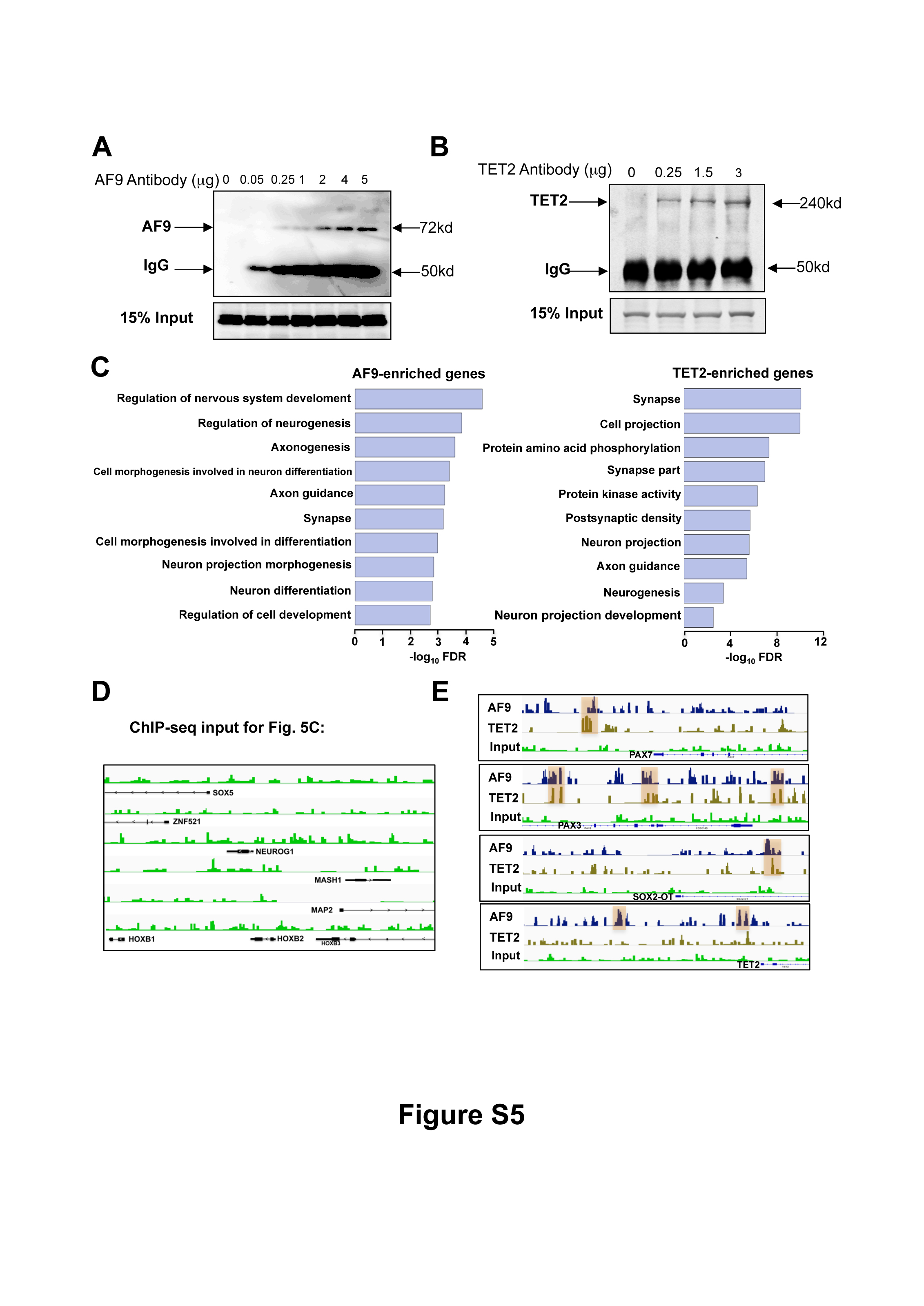

Supplement: Supplementary Figure S5 [file celldisc201517-s6.jpg]

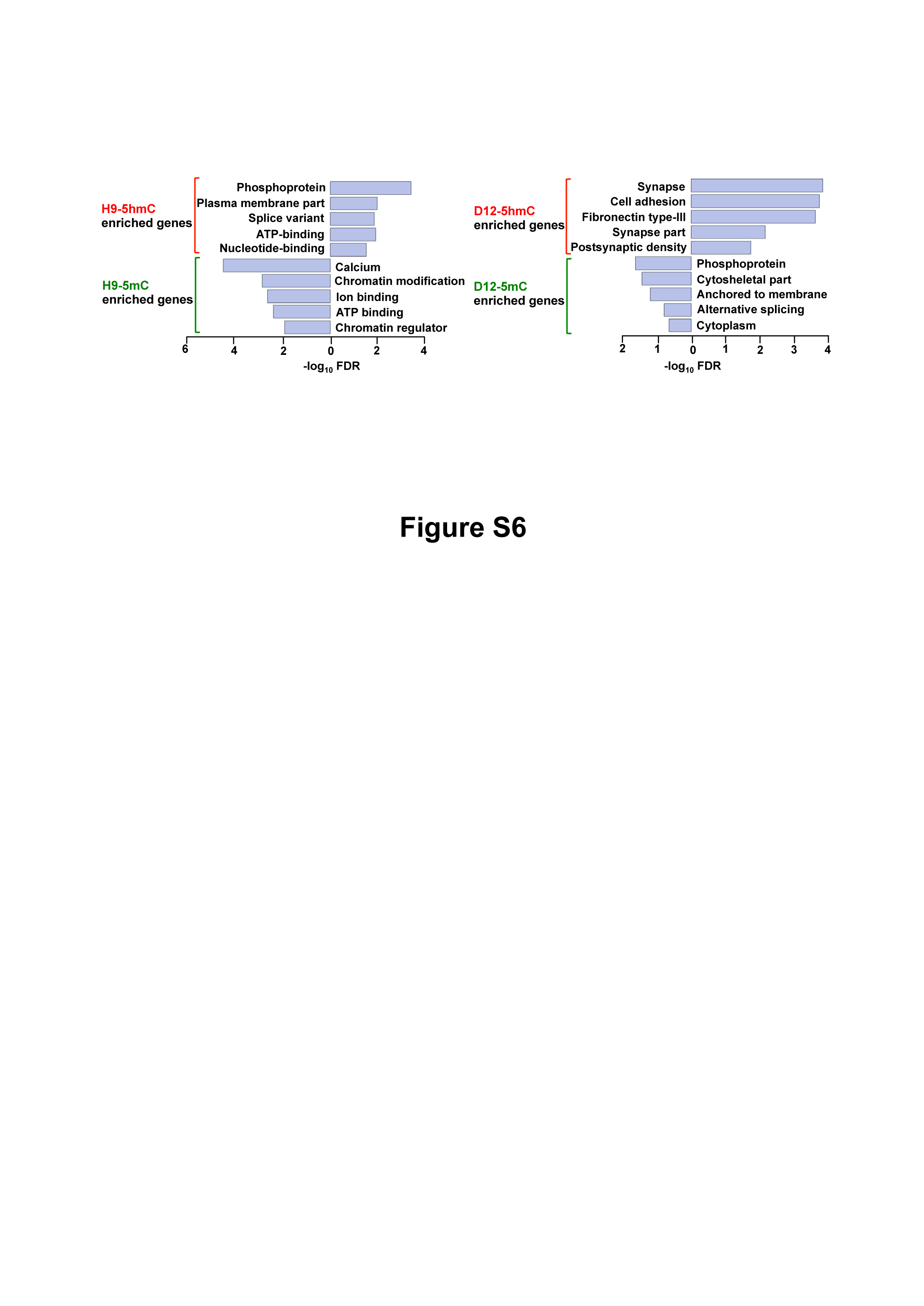

Supplement: Supplementary Figure S6 [file celldisc201517-s7.jpg]

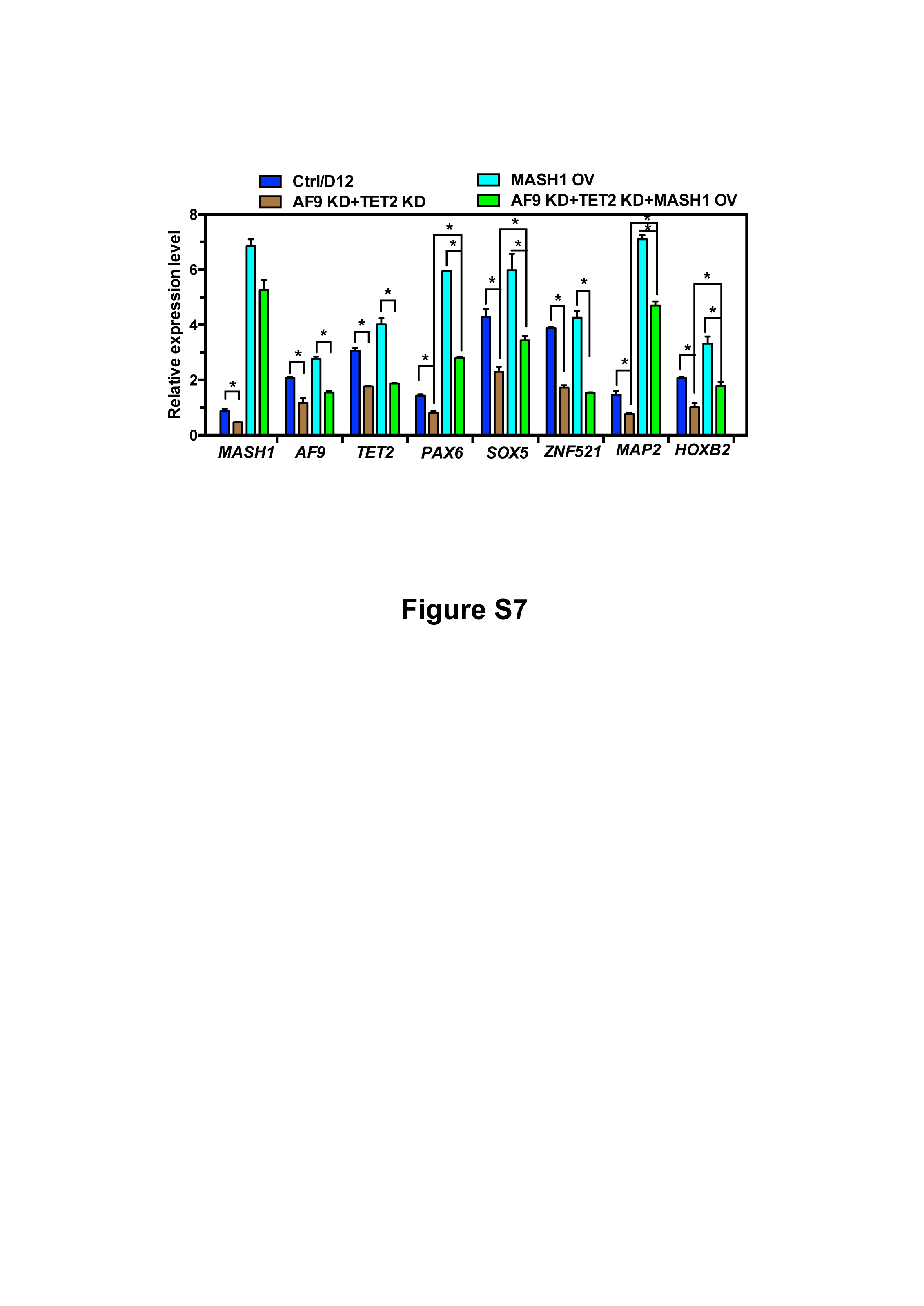

Supplement: Supplementary Figure S7 [file celldisc201517-s8.jpg]
