# Supplementary material for: AF9 promotes hESC neural differentiation through recruiting TET2 to neurodevelopmental gene loci for methylcytosine hydroxylation
Source: Cell Discov. 2015 Jul 28;1:15017–. doi: 10.1038/celldisc.2015.17 (PMC4860857; doi:10.1038/celldisc.2015.17)
Supplement: Supplementary Table S1 [file celldisc201517-s9.pdf]

Accession	Description	Score	Coverage	# Proteins	# Unique Peptides	# Peptides	# PSMs	# AAs	MW [kDa]	calc. pI
HSP70_HUMAN	Heat shock 70 kDa protein 1A/1B	47.28	26.45	28	12	15	36	586	63.9	5.71
	A2	Sequence	# PSMs	# Proteins	# Protein Groups	Protein Group Accessions	Modifications	Δ Cn	q-Value	PEP
	High	NQVALNPQNTVFDAK	3	8	1	HSP70		0.0000	0	0.00102
	High	TTPSYVAFTDTER	5	36	2	E9PKE3;HSP70		0.0000	0	0.006221
	High	DAGVIAGLNVLR	2	15	1	HSP70		0.0000	0	0.02985
	High	YKAEDEVQR	2	19	1	HSP70		0.0000	0	0.02971
	High	ATAGDTHLGGEDFDNR	6	26	1	HSP70		0.0000	0	0.06251
	High	VEIIANDQGNR	1	40	3	E9PKE3;HSP70;P11021		0.0000	0	0.09166
	High	SAVEDEGLK	1	15	1	HSP70		0.0000	0	0.09076
	High	NALESYAFNMK	2	19	1	HSP70		0.0000	0	0.031
	High	FGDPVVQSDMK	2	7	1	HSP70		0.0000	0	0.03113
	High	AQIHDLVLVGGSTR	3	13	1	HSP70		0.0000	0	0.05907
	High	NALESYAFNmK	2	19	1	HSP70	M10(Oxidation)	0.0000	0.002	0.1321
	High	KFGDPVVQSDMK	1	7	1	HSP70		0.0000	0.002	0.1645
	High	LSKEEIER	2	19	1	HSP70		0.0000	0.007	0.2085
	High	AEDEVQR	2	19	1	HSP70		0.0000	0.007	0.2614
	High	ITITNDKGR	1	39	2	E9PKE3;HSP70		0.0000	0.007	0.2712
	High	IINEPTAAAIAYGLDR	1	16	1	HSP70		0.0000	0.007	0.2125
TET2_HUMAN	Ten eleven translocation 2	42.02	14.29	7	9	21	92	1165	130.2	7.80
	A2	Sequence	# PSMs	# Proteins	# Protein Groups	Protein Group Accessions	Modifications	Δ Cn	q-Value	PEP
	High	DFTSFSTHNcSGPENPELQILNEQEGK	3	6	1	TET2	C10(Carbamidomethyl)	0.0000	0	0.001022
	High	NPGESSQPNVSDLSDKK	23	6	1	TET2		0.0000	0	0.002799
	High	RNFGVSQER	2	6	1	TET2		0.0000	0	0.01224
	High	NPGESSQPNVSDLSDK	24	6	1	TET2		0.0000	0	7.01538E-05
	High	ESVSSVAQENAVK	4	6	1	TET2		0.0000	0	0.004104
	High	SVFEIcPSPAENNIQGTTK	6	6	1	TET2	C6(Carbamidomethyl)	0.0000	0	0.0004966
	High	TVSEPSLSGLLQIK	3	7	1	TET2		0.0000	0	0.003448
	High	VSPDFTQESR	4	7	1	TET2		0.0000	0	0.01808
	High	AVLmPNGATVSASSVEHTHGELLEK	2	6	1	TET2	M4(Oxidation)	0.0000	0	0.01612
KRT1_HUMAN	Keratin 1	24.70	15.84	11	10	10	20	644	66.0	8.12
	A2	Sequence	# PSMs	# Proteins	# Protein Groups	Protein Group Accessions	Modifications	Δ Cn	XCorr	Probability
	High	GSGGGSSGGSIGGR	3	7	1	KRT1		0.0000	0	0.00888
	High	YEELQITAGR	4	8	1	KRT1		0.0000	0	0.02273
	High	IEISELNR	3	8	1	KRT1		0.0000	0	0.1006
	High	SLDLDSIIAEVK	1	6	1	KRT1		0.0000	0	0.01632
	High	SLVNLGGSK	3	6	1	KRT1		0.0000	0	0.09819
	High	TLLEGEESR	1	7	1	KRT1		0.0000	0	0.09704
	High	TNAENEFVTIK	1	7	1	KRT1		0.0000	0	0.09712
	High	FLEQQNQVLQTK	2	10	1	KRT1		0.0000	0	0.08885
	High	AQYEDIAQK	1	7	1	KRT1		0.0000	0.007	0.1799
	High	SISISVAR	1	7	1	KRT1		0.0000	0.007	0.2607
P62258_HUMAN	14-3-3 protein epsilon	22.11	30.98	12	6	7	16	255	29.2	4.74
	A2	Sequence	# PSMs	# Proteins	# Protein Groups	Protein Group Accessions	Modifications	Δ Cn	q-Value	PEP
	High	EAAENSLVAYK	3	6	1	P62258		0.0000	0	0.02222
	High	YLAEFATGNDR	3	7	1	P62258		0.0000	0	0.104
	High	AAFDDAIAELDTLSEESYK	2	6	1	P62258		0.0000	0	0.02164
	High	YDEMVESMK	2	6	1	P62258		0.0000	0	0.07925
	High	IISSIEQK	2	8	1	P62258		0.0000	0.002	0.1327
	High	NLLSVAYK	3	34	6	P27348;P62258;P63104;Q4VY20;Q0		0.0000	0.007	0.2581
	High	VAGmDVELTVEER	1	8	1	P62258	M4(Oxidation)	0.0000	0.007	0.1733
MLLT3_HUMAN	Myeloid/lymphoid or mixed-lineage leukemia (trithorax homolog, Drosophila); translocated to, 3	18.31	24.50	1	4	4	6	208	24.2	10.32
	A2	Sequence	# PSMs	# Proteins	# Protein Groups	Protein Group Accessions	Modifications	Δ Cn	q-Value	PEP
	High	GPEHSNIQHFVEK	2	1	1	MLLT3		0.0000	0	0.008934
	High	SAFKEPSRDHNK	1	1	1	MLLT3		0.0000	0	0.05211
	High	AYLDELVELHRR	2	1	1	MLLT3		0.0000	0	0.03487
	High	TNNNQILEVKSPIK	1	1	1	MLLT3		0.0000	0	0.01354
PPM1B_HUMAN	Protein phosphatase 1B (Fragment)	16.27	20.37	10	7	7	13	378	41.8	5.35
	A2	Sequence	# PSMs	# Proteins	# Protein Groups	Protein Group Accessions	Modifications	Δ Cn	q-Value	PEP
	High	GPTEQLVSPEPEVYEILR	1	8	1	PPM1B		0.0000	0	0.04478
	High	SGSALELSVENVK	4	9	1	PPM1B		0.0000	0	0.08317
	High	NVIEAVYSR	3	9	1	PPM1B		0.0000	0	0.08508
	High	SGEEGMPDLAHVMR	2	9	1	PPM1B		0.0000	0	0.09628
	High	VSDEAVKK	1	9	1	PPM1B		0.0000	0.002	0.1704
	High	VEEIMEK	1	9	1	PPM1B		0.0000	0.007	0.227
	High	ALGDYDYK	1	9	1	PPM1B		0.0000	0.007	0.2451
B7Z4V2_HUMAN	cDNA FLJ51907, highly similar to Stress-70 protein	16.12	15.64	10	10	10	16	665	72.4	5.94
	A2	Sequence	# PSMs	# Proteins	# Protein Groups	Protein Group Accessions	Modifications	Δ Cn	q-Value	PEP
	High	TTPSVVAFTADGER	4	6	1	B7Z4V2		0.0000	0	0.004538
	High	VLENAEGAR	2	6	1	B7Z4V2		0.0000	0	0.09674
	High	DAGQISGLNVLR	2	5	1	B7Z4V2		0.0000	0	0.01942
	High	SDIGEVILVGGmTR	1	7	1	B7Z4V2	M12(Oxidation)	0.0000	0	0.04394
	High	AQFEGIVTDLIR	1	7	1	B7Z4V2		0.0000	0.007	0.234
	High	RYDDPEVQK	1	5	1	B7Z4V2		0.0000	0.007	0.2678
	High	DSETGENIR	1	5	1	B7Z4V2		0.0000	0.007	0.1821
AF4_HUMAN	AF4/FMR2 family member 1 isoform 1	10.42	8.06	1	2	2	2	248	29.2	10.65
	A2	Sequence	# PSMs	# Proteins	# Protein Groups	Protein Group Accessions	Modifications	Δ Cn	q-Value	PEP
	High	RAPPEAPHPGK	1	1	1	AF4		0.0000	0	0.009984
	High	REPGLLPYGSR	1	1	1	AF4		0.0000	0	0.08535
Q9BSV4_HUMAN	SFPQ protein	10.49	6.62	6	3	4	7	634	68.6	8.82
	A2	Sequence	# PSMs	# Proteins	# Protein Groups	Protein Group Accessions	Modifications	Δ Cn	q-Value	PEP
	High	FGQGGAGPVGGQGPR	2	3	1	Q9BSV4		0.0000	0	0.02095
	High	ISDSEGFK	2	5	1	Q9BSV4		0.0000	0	0.08728
	High	AVVIVDDR	1	10	2	H7C367;Q9BSV4		0.0000	0.002	0.1685
	High	YGEPGEVFINK	2	5	1	Q9BSV4		0.0000	0.007	0.2116
P22626-2_HUMAN	Isoform A2 of Heterogeneous nuclear ribonucleoproteins A2/B1 OS=Homo sapiens GN=HNRNPA2B1 - [ROA2_HUMA	7.98	15.84	7	4	5	8	341	36.0	8.65
	A2	Sequence	# PSMs	# Proteins	# Protein Groups	Protein Group Accessions	Modifications	Δ Cn	q-Value	PEP
	High	IDTIEIITDR	2	3	1	P22626-2		0.0000	0	0.007636
	High	GGSDGYGSGR	1	2	1	P22626-2		0.0000	0	0.07087
	High	GGNFGFGDSR	2	2	1	P22626-2		0.0000	0	0.08324
	High	GGGGNFGPGPGSNFR	2	2	1	P22626-2		0.0000	0	0.02333
	High	EDTEEHHLR	1	14	2	P09651-3;P22626-2		0.0000	0.007	0.197
P16403_HUMAN	Histone H1.2	7.95	19.25	10	4	4	10	213	21.4	10.93
	A2	Sequence	# PSMs	# Proteins	# Protein Groups	Protein Group Accessions	Modifications	Δ Cn	q-Value	PEP
	High	GTGASGSFK	2	10	1	P16403		0.0000	0	0.07384
	High	ALAAAGYDVEK	3	9	1	P16403		0.0000	0	0.01016
	High	ASGPPVSELITK	4	6	1	P16403		0.0000	0	0.08625
	High	SGVSLAALK	1	6	1	P16403		0.0000	0	0.08269
B4DMA2_HUMAN	cDNA FLJ54023, highly similar to Heat shock protein HSP 90-beta	6.12	6.56	27	4	4	6	686	79.1	5.02
	A2	Sequence	# PSMs	# Proteins	# Protein Groups	Protein Group Accessions	Modifications	Δ Cn	q-Value	PEP
	High	GVVDSEDLPLNISR	2	21	1	B4DMA2		0.0000	0	0.008669
	High	EDQTEYLEER	2	13	1	B4DMA2		0.0000	0	0.1098
	High	EQVANSAFVER	1	6	1	B4DMA2		0.0000	0.007	0.2228
	High	IDIIPNPQER	1	6	1	B4DMA2		0.0000	0.007	0.2507
P06748-3	Isoform 3 of Nucleophosmin	2.38	8.49	8	2	2	3	259	28.4	4.72
	A2	Sequence	# PSMs	# Proteins	# Protein Groups	Protein Group Accessions	Modifications	Δ Cn	q-Value	PEP
	High	VDNDENEHQLSLR	2	5	1	P06748-3		0.0000	0	0.02589
	High	DSKPSSTPR	1	6	1	P06748-3		0.0000	0.007	0.2397
E9PJD9_HUMAN	60S ribosomal protein L27a	2.29	25.27	6	2	2	2	91	10.1	10.21
	A2	Sequence	# PSMs	# Proteins	# Protein Groups	Protein Group Accessions	Modifications	Δ Cn	q-Value	PEP
	High	TGAAPIIDVVR	1	6	1	E9PJD9		0.0000	0	0.0988
	High	NQSFcPTVNLDK	1	5	1	E9PJD9	C5(Carbamidomethyl)	0.0000	0.007	0.2551
SLC25A6_HUMAN	ADP/ATP translocase 3	1.91	5.06	9	1	1	1	158	17.3	9.74
	A2	Sequence	# PSMs	# Proteins	# Protein Groups	Protein Group Accessions	Modifications	Δ Cn	q-Value	PEP
	High	TAVAPIER	1	9	1	SLC25A6		0.0000	0.002	0.1634
SRSF5_HUMAN	Serine/arginine-rich-splicing factor 5	0.00	7.26	15	1	1	1	124	14.4	10.08
	A2	Sequence	# PSMs	# Proteins	# Protein Groups	Protein Group Accessions	Modifications	Δ Cn	q-Value	PEP
	High	LIVENLSSR	1	15	1	SRSF5		0.0000	0.009	0.2808
E9PPU1_HUMAN	40S ribosomal protein S3	0.00	22.15	17	3	3	3	158	17.4	9.50
	A2	Sequence	# PSMs	# Proteins	# Protein Groups	Protein Group Accessions	Modifications	Δ Cn	q-Value	PEP
	High	AELNEFLTR	1	15	1	E9PPU1		0.0000	0	0.09939
	High	ELAEDGYSGVEVR	1	13	1	E9PPU1		0.0000	0	0.04856
	High	DEILPTTPISEQK	1	8	1	E9PPU1		0.0000	0.002	0.1573
