# Supplementary material for: AF9 promotes hESC neural differentiation through recruiting TET2 to neurodevelopmental gene loci for methylcytosine hydroxylation
Source: Cell Discov. 2015 Jul 28;1:15017–. doi: 10.1038/celldisc.2015.17 (PMC4860857; doi:10.1038/celldisc.2015.17)
Supplement: Supplementary Table S2 [file celldisc201517-s10.pdf]

Table S2. shRNA target sequences.

Name	Sequences
Control shRNA	5′-CTTGTGAAGCTTCTAGGAG-3′
AF9 shRNA1	5′-GCAGCAGATCGTGAACCTTAT -3′
AF9 shRNA2	5′-GGTCCTTTGAGGTCTATAATG -3′
TET2 shRNA1	5′-GGGTAAGCCAAGAAAGAAA-3′
TET2 shRNA2	5’-GCAACAGACTAAGTCCATTCC -3’
TET1 shRNA	5’-CCTTGATAGAATCACTCAGTT-3’
