# Supplementary material for: AF9 promotes hESC neural differentiation through recruiting TET2 to neurodevelopmental gene loci for methylcytosine hydroxylation
Source: Cell Discov. 2015 Jul 28;1:15017–. doi: 10.1038/celldisc.2015.17 (PMC4860857; doi:10.1038/celldisc.2015.17)
Supplement: Supplementary Table S3 [file celldisc201517-s11.pdf]

Table S3. Primer sequences (5'→3') for Q-PCR.

Genes	5' primers	3' primers
OCT4	CGTGAAGCTGGAGAAGGAGAAGCTG	CAAGGGCCGCAGCTTACACATGTTC
NANOG	GTGAACCCGACTGGGAACC	GGCAGGAGAATTTGGCTGGA
PAX6	ACAGTCACAGCGGAGTGAATC	ACTTTTGCATCTGCATGGGTC
AF9	TTTGTGGAGAAAGTCGTCTTCC	GAGGTGATTCACTGGTGGATG
GAPDH	CATGAGAAGTATGACAACAGCCT	AGTCCTTCCACGATACCAAAGT
SOX1	ACCAGGCCATGGATGAAG	CTTAATTGCTGGGGAATTGG
TET1	CATCAGTCAAGACTTTAAGCCCT	CGGGTGGTTTAGGTTCTGTTT
TET2	GATAGAACCAACCATGTTGAGGG	TGGAGCTTTGTAGCCAGAGGT
TET3	GCCGGTCAATGGTGCTAGAG	CGGTTGAAGGTTTCATAGAGCC
SOX5	AGGTTCAGTTGGAGACGATCA	TGGTCGCTTGGAAGACATC
MAP2	CTGCTTTACAGGGTAGCACAA	TTGAGTATGGCAAACGGTCTG
TUJ1	GAGCGGATCAGCGTCTACTAC	CCCCACTCTGACCAAAGATGAA
MASH1	CGTCCTCTCCGGAACTGAT	TCCTGCTTCCAAAGTCCATT
BRN2	CGGCGGATCAAACTGGGATTT	TTGCGCTGCGATCTTGTCTAT
NEUROD1	ATGACCAAATCGTACAGCGAG	GTTCATGGCTTCGAGGTCGT
NEUROD2	TGCTACTCCAAGACGCAGAAG	CACGTAGGACACTAGGTCTGG
NEUROD4	ACCAGGTACTTATGGGATGCT	AAGGCGAGCTTTGGTCATCTT
ZNF521	GGCACTAGATTGTAAGAAGAGGC	CATCCACTCCATCTGTCAGTTG
NEUROG1	GCTCTCTGACCCCAGTAGC	GCGTTGTGTGGAGCAAGTC
HOXB2	CGCCAGGATTCACCTTTCCTT	CCCTGTAGGCTAGGGGAGAG
NEUROG1	GCGGATGTCTCTTGGTCTG	AAAAGGAAAGGCCGTCTAGG
OTX2	AGAGGACGACGTTCACTCG	TCGGGCAAGTTGATTTTCAGT
NGN2	TCCTCCGTGTCCTCCAATTC	AGGTGAGGTGCATAGCGGT
GDF3	TCCCGAGACTTATGCTACGTAAA	TTCTTTGGGTAAAGAAAGAAACCTT
CK14	CATGAGTGTGGAAGCCGACAT	GCCTCTCAGGGCATTCATCTC
T	TTCATAGCGGTGACTGCTTATCA	CACCCCCATTGGGAGTACC
SOX7	GAGCTGTCGGATGGACAATCG	CCGTTTCCTCTCGTCCTTGG

SOX17	GTGGACCGCACGGAATTTG	GGAGATTCACACCGGAGTCA
FOXA2	GGAGCAGCTACTATGCAGAGC	CGTGTTCATGCCGTTCATCC
EOMES	GCCGACAATAACATGCAGGG	GTGGGAACCAGTATTAGGAGACT
