# Supplementary material for: AF9 promotes hESC neural differentiation through recruiting TET2 to neurodevelopmental gene loci for methylcytosine hydroxylation
Source: Cell Discov. 2015 Jul 28;1:15017–. doi: 10.1038/celldisc.2015.17 (PMC4860857; doi:10.1038/celldisc.2015.17)
Supplement: Supplementary Table S4 [file celldisc201517-s12.pdf]

**Table S4. Primers for ChIP verification and Glu-qPCR.**

| Name | 5' primers | 3' primers |
|---|---|---|
| *SOX5* R1 | GGGCAGAATATGGCCCAAACAC | ATTCTAACAGGCAGGGTGATGTC |
| *SOX5* R2 | CAGCCTCAGTTAGCAACTTGTTAGT | GTGATTGCAGTGGGAGAGTACTGAC |
| *SOX5* R3 | CATACCTAAGCTATATGGCGCAGC | GCTCTTCCATATACAGCCATATACTG |
| *ZNF521* R1 | GTGAGGTCTCGGCTCACTGCAA | TGTGTAATTGGCTGGGTGCGGTG |
| *ZNF521* R2 | CAGTCCCCATGGCCCACATCCA | AGCCTCATGCGTGGCCCTTGTG |
| *ZNF521* R3 | ATCTCCAGCATCAAGAAGCTGCAG | GCTTAAATTCTCACCCACTCTTGGTAT |
| *NEUROG1* R1 | AGGGTTCAGGGCTCCCAGCAAG | CAAGTACCCTGTCTCAGCATGTC |
| *NEUROG1* R3 | CCGATGTGAATACCTAACTCCAG | CCACTTCAGTAGAGGAGCAGC |
| *MASH1* R1 | TAGTCCAGGCGTGGTGGTTCATG | GTGACGTGATCTCGGCTCACTGC |
| *MASH1* R3 | GAGCGTCTCATAATTTCCAACAGAGC | TCCTCCACACCTAAATTCAGTAGCG |
| *MAP2* R1 | GGCAGTTGTCTACACCTGACCCT | GACCTGATAGATTAGACCTTCCAG |
| *MAP2* R3 | CGTATACTGCTCAAGTGATGGGTG | GTGAGATTGAGGACGGTGTATACTG |
| *HOXB* R1 | GACAGAGTCTTGCTCTGTCACC | GTGGCTCATGCCTGTAATACCAGC |
| *HOXB* R2 | CACTTCTGAATACTGATCTAACTAGG | GAGGAGGTCTTGCAGTGTTGC |
| *HOXB* R3 | GAACAGTGGCACCAAACTATTGTC | TCACAAAGCCAGGATGGTCTCA |
