# Supplementary material for: AF9 promotes hESC neural differentiation through recruiting TET2 to neurodevelopmental gene loci for methylcytosine hydroxylation
Source: Cell Discov. 2015 Jul 28;1:15017–. doi: 10.1038/celldisc.2015.17 (PMC4860857; doi:10.1038/celldisc.2015.17)
Supplement: Supplementary Information [file celldisc201517-s13.pdf]

SUPPLEMENTAL EXPERIMENTAL PROCEDURES

Immunostaining

Immunostaining was performed as described previously (Zhang et al., 2010). The following antibodies were used: anti-TUJ1 polyclonal (1:500, Covance), anti-TUJ1 monoclonal (1:500, Covance), anti-MAP2 (1:500, Sigma), anti-AF9 (1:200, Novus), anti-MASH1 (1:50, Santa Cruz Biotechnology), anti-TET2 (1:100, Abiocode), anti-Sox1 (1:200, Millipore), anti-NeuN (1:50, Millipore) and anti-5hmC (1:1000, Millipore). Specific for 5hmC staining, the PFA (4%) fixed monolayer cells were treated with 0.3% TritonX-100 for 15 min. Then it was incubated with 2 N HCl for 15 min at room temperature and neutralized with 10 mM Tris-Cl (pH 8.0) for 10 min. Subsequently; the cells were subjected for routine blocking and antibody incubation procedures.

Western blot Analysis

Western blot analysis was performed according to our published protocol (Jin et al., 2009). The following antibodies were used: anti-AF9 (1:1000, Novus), anti-TET2 (1:500, Abiocode), anti-RFP (1:1000, Abcam), anti-Myc (1:1000, Santa Cruz), anti-Flag (1:5000, Sigma) and anti-His (1:5000, Sigma).

Microarray Assay and RNA-seq

Cells derived from mock, control or AF9 OV neural-differentiated hESCs were collected in Trizol reagent. The microarray, RNA-seq and data analysis were

all performed by ShanghaiBio Corporation (China, SBC, http://www.shbiotech.org). For RNA-seq analysis, raw reads were mapped using the TopHat version 1.4.1 program (Trapnell et al., 2009). FPKM (fragment per kilo base per million) was assigned as an expression value for each gene using Cufflinks version 1.3.0 software (Trapnell et al., 2010). Then, Cuffdiff software was applied to identify differentially expressed genes between experimental and control groups (Trapnell et al., 2013). Differentially expressed gene heat maps were clustered by k-means clustering using the Euclidean distance as the distance and visualized using Java TreeView software (Saldanha, 2004).

ChIP-seq Analysis

ChIP-seq was performed by the Computational Biology Omics Core (CAS-MPG Partner Institute for Computational Biology, China). Data anlysis was mainly referring to the previous reports (Heinz et al., 2010; Zhang et al., 2008). The SOAP version 2.20 alignment tool was used to align ChIP-Seq reads to the human genome (Li et al., 2009). Only reads with less than two mismatches that uniquely mapped to the genome were used in subsequent analyses. Binding peaks for analyzed factors was analyzed by using FindPeaks Homer software (Heinz et al., 2010). The distance from the peak centers to the annotated transcription start sites (TSS) was calculated and the nearest genes (within ±5 kb flanking peaks) were defined as peak-related

genes.

DIP-seq Analysis

DIP-seq data were displayed as previously described (Shen et al., 2013). Briefly, the raw data was analyzed using HOMER software (Heinz et al., 2010). ChIP-seq data was plotted on the ±5 kb flanking regions 5 kb of the peak centers to create the heatmaps, and then the heatmap data matrix was generated with Cluster 3.0 and Java Tree View (de Hoon et al., 2004; Saldanha, 2004).

Functional Enrichment Analysis

To investigate the functions of assigned genes, functional enrichment analyses were performed using the Database for Annotation, Visualization, and Integrated Discovery (DAVID).

Statistics

For statistical analysis of immunostaining positive cells, at least 10 independent fields were calculated and the final percentage was obtained from three independent experiments. All data in this paper were repeated for at least three times. GraphPad Prism software was used to determine statistically significant differences. Data were presented as mean ± SEM. Student’s t tests were used to compare the effects of different groups. Differences were

considered statistically significant at * $P < 0.05$.

References for Supplemental Experimental Procedures

de Hoon, M.J., Imoto, S., Nolan, J., and Miyano, S. (2004). Open source clustering software. Bioinformatics *20*, 1453-1454.

Heinz, S., Benner, C., Spann, N., Bertolino, E., Lin, Y.C., Laslo, P., Cheng, J.X., Murre, C., Singh, H., and Glass, C.K. (2010). Simple combinations of lineage-determining transcription factors prime cis-regulatory elements required for macrophage and B cell identities. Molecular cell *38*, 576-589.

Jin, Z., Liu, L., Bian, W., Chen, Y., Xu, G., Cheng, L., and Jing, N. (2009). Different transcription factors regulate nestin gene expression during P19 cell neural differentiation and central nervous system development. The Journal of biological chemistry *284*, 8160-8173.

Li, R., Yu, C., Li, Y., Lam, T.W., Yiu, S.M., Kristiansen, K., and Wang, J. (2009). SOAP2: an improved ultrafast tool for short read alignment. Bioinformatics *25*, 1966-1967.

Saldanha, A.J. (2004). Java Treeview--extensible visualization of microarray data. Bioinformatics *20*, 3246-3248.

Shen, L., Wu, H., Diep, D., Yamaguchi, S., D'Alessio, A.C., Fung, H.L., Zhang, K., and Zhang, Y. (2013). Genome-wide analysis reveals TET- and TDG-dependent 5-methylcytosine oxidation dynamics. Cell *153*, 692-706.

Trapnell, C., Hendrickson, D.G., Sauvageau, M., Goff, L., Rinn, J.L., and Pachter, L. (2013). Differential analysis of gene regulation at transcript resolution with RNA-seq. Nature biotechnology *31*, 46-53.

Trapnell, C., Pachter, L., and Salzberg, S.L. (2009). TopHat: discovering splice junctions with RNA-Seq. Bioinformatics *25*, 1105-1111.

Trapnell, C., Williams, B.A., Pertea, G., Mortazavi, A., Kwan, G., van Baren, M.J., Salzberg, S.L., Wold, B.J., and Pachter, L. (2010). Transcript assembly and quantification by RNA-Seq reveals unannotated transcripts and isoform switching during cell differentiation. Nature biotechnology *28*, 511-515.

Zhang, K., Li, L., Huang, C., Shen, C., Tan, F., Xia, C., Liu, P., Rossant, J., and

Jing, N. (2010). Distinct functions of BMP4 during different stages of mouse ES cell neural commitment. Development *137*, 2095-2105.

Zhang, Y., Liu, T., Meyer, C.A., Eeckhoute, J., Johnson, D.S., Bernstein, B.E., Nusbaum, C., Myers, R.M., Brown, M., Li, W.*, et al.* (2008). Model-based analysis of ChIP-Seq (MACS). Genome biology *9*, R137.
